# Single-dose oral ciprofloxacin prophylaxis as a response to a meningococcal meningitis epidemic in the African meningitis belt: A 3-arm, open-label, cluster-randomized trial

**DOI:** 10.1371/journal.pmed.1002593

**Published:** 2018-06-26

**Authors:** Matthew E. Coldiron, Bachir Assao, Anne-Laure Page, Matt D. T. Hitchings, Gabriel Alcoba, Iza Ciglenecki, Céline Langendorf, Christopher Mambula, Eric Adehossi, Fati Sidikou, Elhadji Ibrahim Tassiou, Victoire De Lastours, Rebecca F. Grais

**Affiliations:** 1 Epicentre, Paris, France; 2 Epicentre, Maradi, Niger; 3 Harvard T.H. Chan School of Public Health, Boston, Massachusetts, United States of America; 4 Médecins Sans Frontières, Geneva, Switzerland; 5 Médecins Sans Frontières, Paris, France; 6 Niamey National Hospital, Niamey, Niger; 7 Centre de Recherche Médicale et Sanitaire, Niamey, Niger; 8 Ministry of Public Health, Madarounfa, Niger; 9 Department of Internal Medicine, Hôpital Beaujon, Assistance Publique–Hôpitaux de Paris, Paris, France; 10 IAME Research Group UMC1137, Université Paris Diderot, Paris, France; Centers for Disease Control and Prevention, UNITED STATES

## Abstract

**Background:**

Antibiotic prophylaxis for contacts of meningitis cases is not recommended during outbreaks in the African meningitis belt. We assessed the effectiveness of single-dose oral ciprofloxacin administered to household contacts and in village-wide distributions on the overall attack rate (AR) in an outbreak of meningococcal meningitis.

**Methods and findings:**

In this 3-arm, open-label, cluster-randomized trial during a meningococcal meningitis outbreak in Madarounfa District, Niger, villages notifying a suspected case were randomly assigned (1:1:1) to standard care (the control arm), single-dose oral ciprofloxacin for household contacts within 24 hours of case notification, or village-wide distribution of ciprofloxacin within 72 hours of first case notification. The primary outcome was the overall AR of suspected meningitis after inclusion. A random sample of 20 participating villages was enrolled to document any changes in fecal carriage prevalence of ciprofloxacin-resistant and extended-spectrum beta-lactamase (ESBL)–producing Enterobacteriaceae before and after the intervention. Between April 22 and May 18, 2017, 49 villages were included: 17 to the control arm, 17 to household prophylaxis, and 15 to village-wide prophylaxis. A total of 248 cases were notified in the study after the index cases. The AR was 451 per 100,000 persons in the control arm, 386 per 100,000 persons in the household prophylaxis arm (*t* test versus control *p* = 0.68), and 190 per 100,000 persons in the village-wide prophylaxis arm (*t* test versus control *p* = 0.032). The adjusted AR ratio between the household prophylaxis arm and the control arm was 0.94 (95% CI 0.52–1.73, *p* = 0.85), and the adjusted AR ratio between the village-wide prophylaxis arm and the control arm was 0.40 (95% CI 0.19‒0.87, *p* = 0.022). No adverse events were notified. Baseline carriage prevalence of ciprofloxacin-resistant Enterobacteriaceae was 95% and of ESBL-producing Enterobacteriaceae was >90%, and did not change post-intervention. One limitation of the study was the small number of cerebrospinal fluid samples sent for confirmatory testing.

**Conclusions:**

Village-wide distribution of single-dose oral ciprofloxacin within 72 hours of case notification reduced overall meningitis AR. Distributions of ciprofloxacin could be an effective tool in future meningitis outbreak responses, but further studies investigating length of protection, effectiveness in urban settings, and potential impact on antimicrobial resistance patterns should be carried out.

**Trial registration:**

ClinicalTrials.gov NCT02724046

## Abstract 2

In a cluster-randomized trail in rural Nigerian villages, Matthew E Coldiron and colleagues investigate two different arms of chemoprophylaxis for meningococcal meningitis compared to standard care during a meningitis epidemic.

## Author summary

**Why was this study done?:**

- Antibiotic prophylaxis for close contacts of meningococcal meningitis cases is standard during outbreak response outside of the African meningitis belt.
- There is little evidence about the effectiveness of antibiotic prophylaxis during outbreaks in the African meningitis belt, where the epidemiology of meningococcal meningitis is unique.
- The recent emergence *Neisseria meningitidis* serogroup C combined with limited supplies of vaccine against this serogroup mean that new outbreak response strategies are needed.

**What did the researchers do and find?:**

- We performed a cluster-randomized trial during a meningococcal meningitis epidemic in rural Niger comparing attack rates in 3 different groups.
- Villages were randomized to receive no prophylaxis, prophylaxis for household members of suspected meningitis cases, or village-wide prophylaxis after the first suspected case in the village; directly observed single-dose oral ciprofloxacin was used for prophylaxis.
- At the end of the epidemic, compared to the control villages, there was no difference in attack rate in household prophylaxis villages, but the attack rate was 60% lower in villages receiving village-wide prophylaxis.
- We also performed a study to investigate the effects of the village-wide distribution strategy on patterns of antimicrobial resistance; 95% of healthy participants recruited in the community carried ciprofloxacin-resistant bacteria in their stool at baseline, which was a much larger proportion than expected.

**What do the findings mean?:**

- Village-wide distributions of single-dose oral ciprofloxacin reduced meningitis attack rates.
- The low cost and easy storage and administration of ciprofloxacin could make this strategy an important part of epidemic response.
- Further studies are needed to investigate this strategy in other contexts, including in larger epidemics earlier in the meningitis season, as well as epidemics in urban areas.
- Future studies will also need to continue evaluating the impact of this strategy on antimicrobial resistance patterns.

## Introduction

Cyclical, seasonal epidemics of meningococcal meningitis have been described for at least the past century in a region of sub-Saharan Africa called the “meningitis belt” [1]. The largest epidemics, sometimes with over 100,000 cases, have been due to *Neisseria meningitidis* serogroup A (NmA) [2], but sizeable epidemics have also been caused by serogroups W and X. Epidemics of all 3 of these serogroups have been reported in Niger [3–5]. The introduction of an effective and affordable conjugate vaccine against NmA, PsA-TT (MenAfriVac, Serum Institute of India, Pune, India), beginning in 2010, dramatically reduced incidence and nasopharyngeal carriage of NmA, virtually eliminating it as a public health problem in the meningitis belt [6].

In 2013 and 2014, localized epidemics of meningitis due to a novel strain of *N*. *meningitidis* serogroup C (NmC) occurred in northwestern Nigeria, the first outbreaks of NmC described in Africa since 2 small outbreaks in the 1970s [7]. In 2015, over 15,000 cases, predominantly of NmC, were reported in Niger and Nigeria [8]. The emergence of this strain is seen as a natural evolutionary occurrence in the population of *N*. *meningitidis*, as opposed to PsA-TT-related serogroup replacement [9].

Response to meningococcal meningitis outbreaks is based on enhanced surveillance and biological confirmation, case management, and reactive vaccination, generally with polysaccharide vaccines, which do not clear nasopharyngeal carriage of meningococci and confer protection for only a few years [10]. International stockpiles of meningococcal vaccines are dispatched by the International Coordinating Group, a group of intergovernmental and nongovernmental organizations hosted by the World Health Organization (WHO) whose mission is to supply vaccines in response to disease outbreaks. Nonetheless, there are often significant delays in reactive vaccination, decreasing its potential impact [11]. Decreasing the time to vaccination would likely be beneficial [12], but is logistically difficult in the context of many meningitis belt countries. Further complicating the current situation is a shortage of available vaccines, particularly against NmC.

Since the beginning of the antibiotic era, chemoprophylaxis of close contacts of meningitis cases has been standard in high-resource settings, with the goal of clearing nasopharyngeal carriage of the meningococcus [13]. Meta-analysis of data from high-resource settings showed that prophylaxis of household contacts was associated with an 84% reduction in the risk of meningitis [14]. In the meningitis belt, data are scarce, but 1 quasi-randomized study in Sudan in 1953 showed a significant reduction in meningitis incidence after mass prophylaxis with either sulfadimidine or penicillin G [15]. Historically, as meningococci developed resistance to sulfa derivatives, and as polysaccharide vaccines became available, use of prophylaxis during epidemics disappeared in the meningitis belt. In the most recent WHO recommendations, prophylaxis of household contacts of cases is recommended during non-epidemic periods, but during epidemics, it is not recommended, both because of lack of evidence of benefit and also because of concerns about logistic and operational constraints during the acute phase of an epidemic [16]. But given the epidemiology of the meningitis belt, where low humidity, timing of the first rainfall, and highly variable nasopharyngeal carriage rates lead to different transmission dynamics [17], focusing only on households might not change the course of an epidemic.

After the emergence of NmC, and in the presence of an insufficient quantity of vaccine against this serogroup, a WHO expert panel recommended a trial of antibiotic prophylaxis with ciprofloxacin for household contacts of meningitis cases during an outbreak in the meningitis belt [18]. The objective of our study was thus to evaluate the effect of ciprofloxacin prophylaxis on overall meningitis attack rates (ARs).

## Methods

### Study design

A 3-arm, open-label, cluster-randomized trial was designed for implementation during a meningococcal meningitis epidemic to evaluate community-level meningitis ARs, thus justifying cluster-level randomization. Predefined criteria for study launch included having at least 2 health areas (HAs) of a district cross the WHO meningitis epidemic threshold in the same week (see below for description of administrative divisions in Niger). All villages in a HA that had passed the weekly epidemic threshold during any week after study initiation were eligible for inclusion. The threshold was an AR of 10 suspected cases per 100,000 persons per week in HAs with population ≥ 30,000 or 5 suspected cases per week (total) in HAs with population < 30,000 [16]. Villages were randomized and received their trial intervention once the first suspected case was reported in each village after study launch. In villages, all individuals were eligible to participate in the study, as detailed below.

A cluster was defined as a village. Village chiefs were asked to provide written permission for their village’s participation in the study, but were not told of the village’s treatment assignment beforehand. Village chiefs were “gatekeepers” as set forth by the Ottawa Statement on the Ethical Conduct of Cluster Randomized Trials, and established criteria for the waiver of individual-level informed consent were met [19]. The study protocol (S1 Protocol) was reviewed and approved by the National Consultative Ethics Committee of Niger (Ref: 003/2016/CCNE) and the Ethics Review Board of Médecins Sans Frontières (Ref: 1603). Full trial methods have been previously published [20], and a CONSORT checklist is available (S1 CONSORT Checklist). The trial is registered at ClinicalTrials.gov (NCT02724046).

### Study setting

The study took place in the Madarounfa District, in rural southern Niger (estimated population 518,870), which is divided into 25 HAs, each of which has 1 health center (HC). Two of the 25 HCs have a full-time physician, and the others are staffed with a full-time nurse. There is 1 district hospital with limited laboratory and surgical capacities. Prior to 2017, the most recent meningitis epidemic in the area occurred in 2009 and was due to NmA.

### Randomization and masking

Villages were randomized in a 1:1:1 ratio to receive 1 of the 3 interventions: standard care (the control arm), ciprofloxacin prophylaxis for household members of suspected cases, or village-wide prophylaxis after notification of the first suspected case in a village. Prior to the epidemic, it was impossible to know which villages would notify suspected cases, so a full randomization list based on village names was not prepared in advance. In order to ensure balanced randomization over an unknown epidemic length and between different HAs, a random sorting algorithm was used to create lists for each HA, with a 10% maximum allowable deviation between groups at any point. Sealed opaque envelopes were used to conceal group allocation. Reference lists of included villages and their allocations were kept both centrally and in each HC. All trial participants and investigators were aware of group assignment.

### Meningitis surveillance

Study staff identified suspected cases of meningitis through passive surveillance at HCs using standard WHO case definitions. A suspected case of meningitis was defined as any person with sudden onset of fever (>38.5°C rectal or 38.0°C axillary) and 1 of the following signs: neck stiffness, altered consciousness, or other meningeal sign [16]. A confirmed case was a suspected case with meningococcus detected in the cerebrospinal fluid (CSF) by PCR. Independent of the trial, all case patients received standard care for meningitis, including a 5-day course of ceftriaxone. When lumbar puncture was performed, CSF was collected in sterile vials at the HC and sent to the district hospital for dispatch to Niamey to the Centre de Recherche Médicale et Sanitaire (CERMES), the national reference laboratory for meningitis, for PCR testing [21]. Case notification forms were completed in duplicate and sent with all samples, following national procedures. A third copy of the form was kept on-site for all cases notified from villages included in the study. This form contained demographic and clinical information, including PCR results, and was modified to capture information about receipt of ciprofloxacin and served as the data source for cases included in the study.

### Study procedures

In the control arm, after inclusion of a village, a nurse visited the village and provided information about signs and symptoms of meningitis to village leaders. These informational visits were also carried out in the other 2 study arms. Similar dedicated health education activities are a standard part of epidemic response in the meningitis belt.

In the household prophylaxis arm, directly observed single-dose oral ciprofloxacin was offered to household members of suspected cases within 24 hours of notification, with all doses taken at the same time. A household was defined as a group of people living in the same building or compound under the authority of a single head of household. When subsequent suspected cases were notified from a village randomized to this arm, their households were offered ciprofloxacin prophylaxis, but a given household was eligible to receive ciprofloxacin only once during the course of the epidemic.

In the village-wide prophylaxis arm, a mass distribution of directly observed single-dose oral ciprofloxacin was organized in the village within 72 hours of inclusion. This distribution took place at a fixed site or during door-to-door visits, depending on local preferences. Each distribution lasted 1 day. A village could receive only 1 mass distribution over the course of the epidemic.

Ciprofloxacin oral suspension (Bayer, Leverkusen, Germany) and tablets (Remedica, Limassol, Cyprus) were administered on an age-based scale (S1 Table). All doses were directly observed by a nurse or nurse’s aide, and no doses of ciprofloxacin were left behind in the community or with family members. Tally sheets were used to record the number of doses of ciprofloxacin administered in both the household and village-wide prophylaxis arms. An exhaustive door-to-door census was performed in each village.

All persons living in a household or village randomized to receive ciprofloxacin were offered prophylaxis except persons with a known allergy to ciprofloxacin and persons who showed any sign of meningitis. Persons with suspected meningitis were referred immediately and free of charge to the nearest HC.

Passive serious adverse event surveillance was implemented at all distribution sites and at each HC in included HAs. Serious adverse events were defined following the Brighton Collaboration standards [22].

Senior study staff were identified prior to the epidemic season, and were on standby to implement the trial. Additional study staff working in the field (nurses, nurse’s aides, and laboratory technicians) were identified prior to the epidemic, and brought online as study activities began. All field staff received training on study standard operating procedures prior to carrying out any study activities, including performing censuses, distributing ciprofloxacin, performing meningitis and adverse event surveillance, and collecting CSF. All study materials were procured prior to the beginning of the epidemic season, and stored under manufacturer’s recommended conditions at Epicentre’s research center in Maradi, Niger.

### Outcomes and statistical analysis

The study interventions were offered at the cluster level, and the prespecified primary outcome measure, overall AR of suspected meningitis during the epidemic after inclusion of a village, was assessed at the cluster level from the day of the village’s inclusion until the end of the epidemic. Prespecified secondary outcomes included AR by age and sex, which were assessed at the cluster level, and also the individual protective effectiveness against suspected meningitis conferred by single-dose oral ciprofloxacin, which was assessed at the individual level.

Because multiple factors, such as distribution of village size and overall amplitude of the epidemic, could not be reliably estimated in advance, sample size was not set a priori [23]. A set of contingency tables was prepared, to show necessary sample size assuming 50%, 70%, and 90% AR reduction between each intervention arm and the control arm with 90% power and alpha-error of 5%, given different overall AR values and assuming an intra-cluster correlation coefficient (ICC) of 0.025 [24,25]. It was planned to set a final target sample size after 4 weeks of accrued trial time based on the observed AR and the distribution of village populations [26], but this was not possible because of the late onset of this epidemic during the epidemic season. The epidemic had effectively ended 4 weeks after the trial start.

For the main analysis, to calculate AR, we included the entire population of all included villages, excluding only the index case triggering inclusion in the trial from both the numerator and denominator. Given that the objective of the trial was to test the effectiveness of prophylaxis as a response strategy, taking into account the inherent delay between case notification and interventions, all suspected cases notified after inclusion of a village were included in AR calculations, even if they were notified prior to distribution of ciprofloxacin. We assessed the sensitivity of the primary analysis to exclusion of suspected cases notified between randomization and the study intervention. To assess the difference in meningitis incidence between the 3 arms, we used a cluster-level *t* test of the log-transformed ARs, using inverse variance weights to account for different cluster sizes and numbers of cases. We compared each treatment arm pairwise to the control arm, at a significance level of 5%. After exclusion of index cases, villages with an AR of 0 cases (which could not be log-transformed) were assigned a small number of cases (c < 1) prior to log transformation, and the effect of this parameter was tested in a sensitivity analysis. The validity of the assumptions for the *t* test (in particular, whether log-transformed ARs were approximately normally distributed) was assessed by inspection of a Q-Q plot, and a *p-*value was obtained using the Shapiro–Wilk test applied to the residual log-transformed ARs [27].

The following variables were measured and assessed for imbalance between the 3 arms using a *t* test for continuous variables and a Pearson chi-squared test of independence for categorical variables: proportion of the village under 30 years old, proportion of the village that was male, number of days from inclusion to when the village was targeted for vaccination, number of days between the start of the epidemic and inclusion, and whether inclusion of the village occurred after the first day of rainfall. To control for potential confounders that were imbalanced between the arms, we performed a Poisson regression to estimate AR ratios and 95% confidence intervals associated with each treatment arm compared to the control arm. We corrected the standard errors to account for overdispersion in the data, i.e., when the variance in ARs was greater than the mean. The original study protocol (S1 Protocol) stated that the comparison of adjusted ARs would be made with ANOVA or multiple linear regression. During the review process for publication of the study protocol [20], it was suggested that the use of Poisson regression with overdispersion would be more appropriate. This change was made in the published protocol and the statistical analysis plan. The ICC was calculated using the method described by Yelland and colleagues [28].

Subgroup analysis by age and sex was performed using the same methods as above on a subset of the suspected cases and villages, as was analysis of PCR-confirmed meningitis. The individual-level association between taking ciprofloxacin and meningitis incidence was estimated using mixed-effects logistic regression to account for clustering of cases by village, used data on ciprofloxacin coverage from distribution tally sheets, and assumed that all cases of meningitis were notified in the HCs.

Data are deposited in the Dryad repository: http://dx.doi.org/10.5061/dryad.83576bv [29]. Data were analyzed with SAS version 9.4.

### Antibiotic resistance sub-study

Ten villages in the control arm and 10 villages in the village-wide prophylaxis arm were randomly selected to participate in a sub-study examining fecal carriage of ciprofloxacin-resistant and extended-spectrum beta-lactamase (ESBL)–producing Enterobacteriaceae. Using information collected during the village census, 20 households were randomly selected in each participating village. After arrival of the study staff in the household, 1 household member was chosen by drawing lots. Written informed consent was obtained from individual participants in the sub-study.

Stool samples were collected at days 0 (prior to ciprofloxacin administration in the village-wide prophylaxis arm), 7, and 28. Samples were inoculated into Cary–Blair media (Copan, Brescia, Italy) in the participants’ homes and transported to the laboratory in Maradi, where they were plated on MacConkey agar media (Oxoid, Basingstoke, UK) containing 1 mg/l ciprofloxacin alone, 1 mg/l cefotaxime alone, and 1 mg/l of both ciprofloxacin and cefotaxime. For the first 100 samples processed, colonies growing on the antibiotic-impregnated agar were identified using standard microbiological methods. The presence of ESBL-producing bacteria was confirmed by the synergy test, and minimum inhibitory concentration to ciprofloxacin was determined using the E-test. As the presence of ESBL and resistance to ciprofloxacin were confirmed for all colonies growing on the corresponding medium, colony characterization was stopped after the first 100 samples, and thereafter all colonies growing on the antibiotic-impregnated agar were considered resistant, but not speciated. For quality control, 10% of all samples were sent to the IAME laboratory at the Université Paris Diderot, a reference laboratory where identification, full antibiotic susceptibility testing, and presence of ESBL were confirmed by mass spectrometry (MALDI-TOF), the disk diffusion method, and molecular methods, respectively [30]. Antibiotic susceptibility testing was performed and interpreted using the European Committee on Antimicrobial Susceptibility Testing guidelines [31].

To assess the effect of village-wide distributions of ciprofloxacin on prevalence of fecal carriage of ciprofloxacin-resistant and/or ESBL-producing Enterobacteriaceae over time, we performed a linear regression, adjusting for age and sex of the individuals and using the generalized estimating equation method to account for village- and individual-level clustering.

## Results

### Timeline

**Fig 1:**
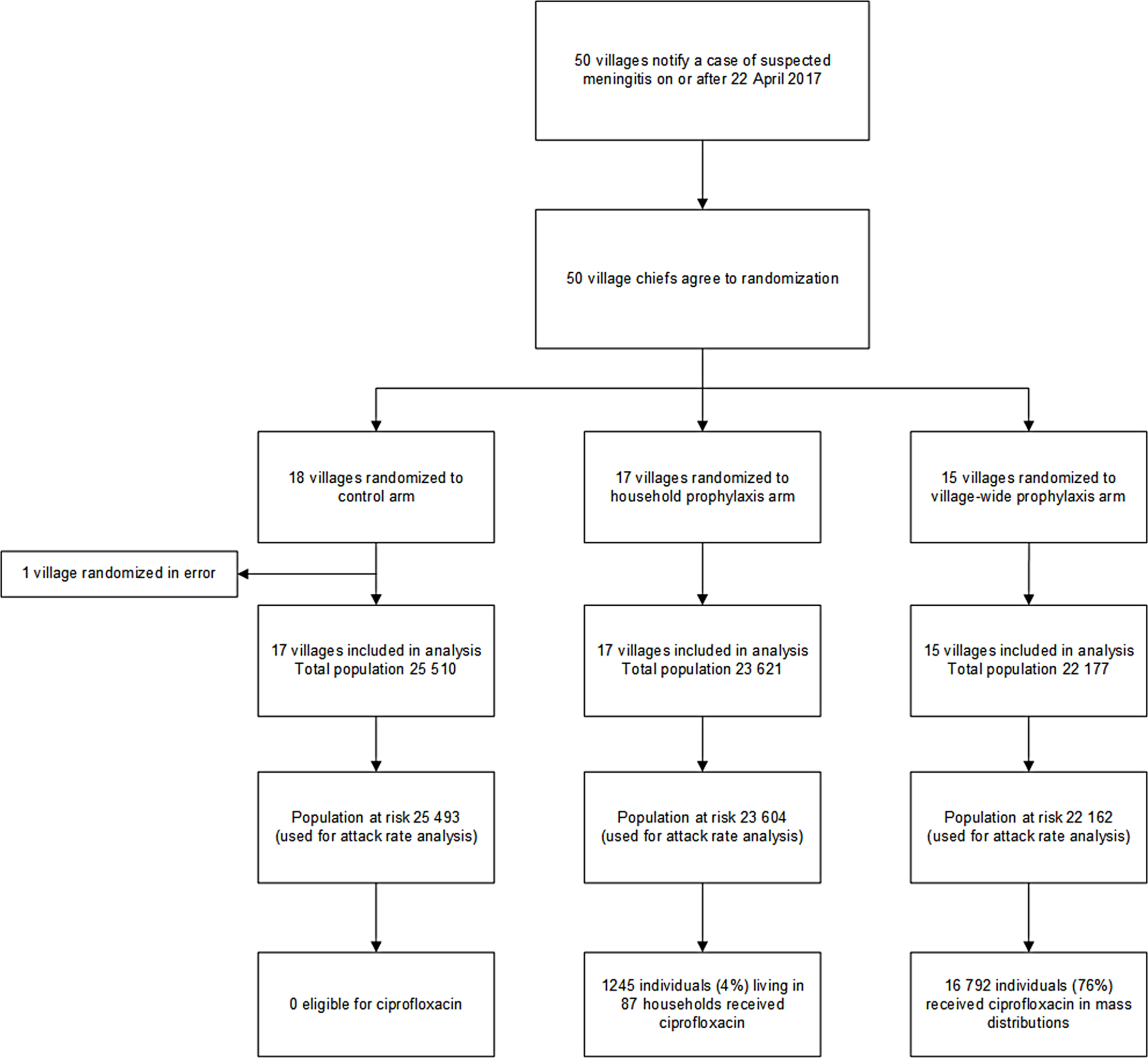
Study flow, Madarounfa District, Niger, 2017.

**Fig 2:**
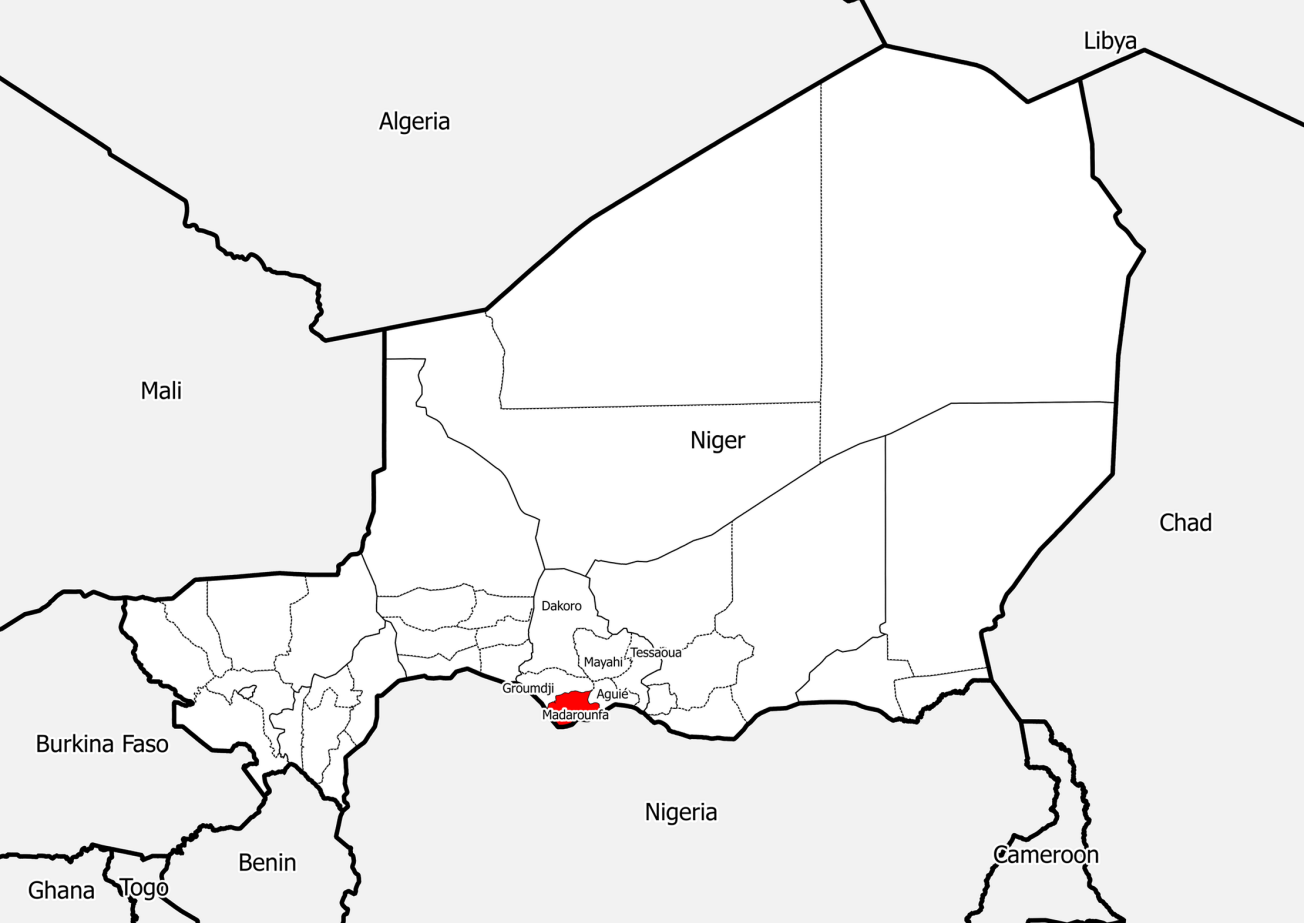
Madarounfa District, Niger.

On April 20, 2017, the Madarounfa District in the Maradi Region of Niger met preestablished study start criteria, as 2 HAs crossed the weekly epidemic threshold during the same week. The trial began on April 22, 2017, and over the course of the epidemic, 50 villages in a total of 5 HAs were enrolled (Figs 1 and 2); 1 village was enrolled in error. The final village was enrolled on May 18, 2017, and HC-based surveillance continued through May 31, 2017. The final case was notified on May 23, 2017. All eligible villages notifying a suspected case of meningitis were included in the study. The first rain of the season fell in the study area on May 10, 2017. Independent of the functioning of the trial, a reactive vaccination campaign with an AC polysaccharide vaccine was carried out by the Ministry of Public Health in the study area between May 12 and 18, 2017, targeting 47 of the 49 villages included in the trial.

### Main trial results

The total population of the villages included in the trial was 71,308, and their demographic structures were similar across arms (Table 1). The age and sex of suspected meningitis cases notified were similar across the 3 arms. Timing between inclusion in the study and reactive vaccination was also similar across the 3 arms. In the village-wide prophylaxis arm, the median coverage of the ciprofloxacin distributions per village was 77% (IQR 75%‒81%, range 56%‒100%), and the mean time from randomization to ciprofloxacin distribution was 2.4 days (SD 0.5). In the household prophylaxis arm, the median proportion of a village’s population that received ciprofloxacin was 4% (IQR 2%‒6%, range 0.2%‒15%).

**Table 1:** Baseline characteristics of villages.

Characteristic	Control (*n* = 17 villages; population = 25,510)	Household prophylaxis (*n* = 17 villages; population = 23,621)	Village-wide prophylaxis (*n* = 15 villages; population = 22,177)
**Sex, *n* (%)**			
Male	12,473 (49%)	11,477 (49%)	10,889 (49%)
Female	13,037 (51%)	12,144 (51%)	11,288 (51%)
**Village population, median (IQR)**	1,135 (903‒1,594)	1,169 (716‒2,045)	1,399 (924‒1,879)
**Total population <30 years, *n* (%)**	19,748 (77%)	18,293 (77%)	17,031 (76%)
**Villages targeted by vaccination campaign, *n* (%)**	17 (100%)	16 (94%)	14 (93%)
**Days between inclusion and vaccination, mean (SD)**	11.5 (7.8)	10.8 (9.5)	12.2 (8.8)
**Days between inclusion and first rainfall, mean (SD)**	7.8 (6.9)	6.4 (8.1)	7.1 (6.5)
**Case triggering inclusion of village**			
Age in years, mean (SD)	14.5 (13.0)	11.0 (11.2)	21.4 (19.9)
Sex, *n/N* (%)			
Male	8/17 (47%)	8/17 (47%)	7/15 (47%)
Female	9/17 (53%)	9/17 (53%)	8/15 (53%)
Days between symptom onset and consultation, mean (SD)	1.4 (1.2)	1.9 (1.5)	1.9 (2.6)
**All cases notified in village**			
Age in years, mean (SD)	17.8 (12.6)	17.1 (14.9)	17.8 (17.3)
Sex, *n/N* (%)			
Male	55/132 (42%)	48/108 (44%)	28/57 (49%)
Female	77/132 (58%)	60/108 (56%)	29/57 (51%)
Days between symptom onset and consultation, mean (SD)	1.1 (1.1)	1.3 (1.3)	1.3 (1.6)

A total of 297 suspected cases of meningitis were notified in the study area during the study period, including the 49 that triggered village inclusion and 248 thereafter: 115 in the control arm (AR 451 per 100,000 persons), 91 in the household prophylaxis arm (AR 386 per 100,000 persons), and 42 in the village-wide prophylaxis arm (AR 190 per 100,000 persons). The log-transformed incidence rates displayed reasonable agreement with the normal distribution on the Q-Q plot and significance test (*p-*value from Shapiro–Wilk test: 0.31). There was a difference in the AR between the village-wide prophylaxis arm and the control arm (*t* test *p-*value: 0.032), but not between the household prophylaxis arm and the control arm (*t* test *p-*value: 0.68). These results were not sensitive to the choice of constant c used to account for villages with 0 cases after inclusion. In adjusted analysis, the only confounder retained in the model was whether a village was included before or after the first rainfall. Comparing the village-wide prophylaxis arm with the control arm, the adjusted AR ratio was 0.40 (95% CI 0.19‒0.87, *p* = 0.022), and comparing the household prophylaxis arm to the control arm, the adjusted AR ratio was 0.94 (95% CI 0.52‒1.73, *p* = 0.84) (Table 2). The ICC for the primary outcome was 0.00258. After excluding cases occurring between randomization of a village and the intervention in a village, the adjusted AR ratios were 0.35 (95% CI 0.15–0.80, *p* = 0.01) for the village-wide prophylaxis arm and 0.82 (95% CI 0.43–1.55, *p* = 0.54) for the household prophylaxis arm.

**Table 2:** Attack rate by study intervention.

Treatment arm	Number of cases	AR per 100,000 persons (95% CI)	Crude ARR (95% CI), *p-*value	Adjusted ARR* (95% CI), *p-*value
Control	115	451.0 (262.2‒776.1)	1	1
Household prophylaxis	91	385.5 (224.5‒662.0)	0.85 (0.42‒1.75) *p* = 0.67	0.94 (0.52‒1.73) *p* = 0.85
Village-wide prophylaxis	42	189.5 (98.8‒363.5)	0.42 (0.17‒1.06) *p* = 0.07	0.40 (0.19‒0.87) *p* = 0.022

*Adjusted for whether village was included after the first day of rainfall (May 10, 2017).

AR, attack rate; ARR, attack rate ratio.

In the control arm, the mean number of suspected cases per village was 6.8 (SD 9.7), in the household prophylaxis arm, it was 5.4 (SD 9.7), and in the village-wide prophylaxis arm, it was 2.8 (SD 5.6).

**Fig 3:**
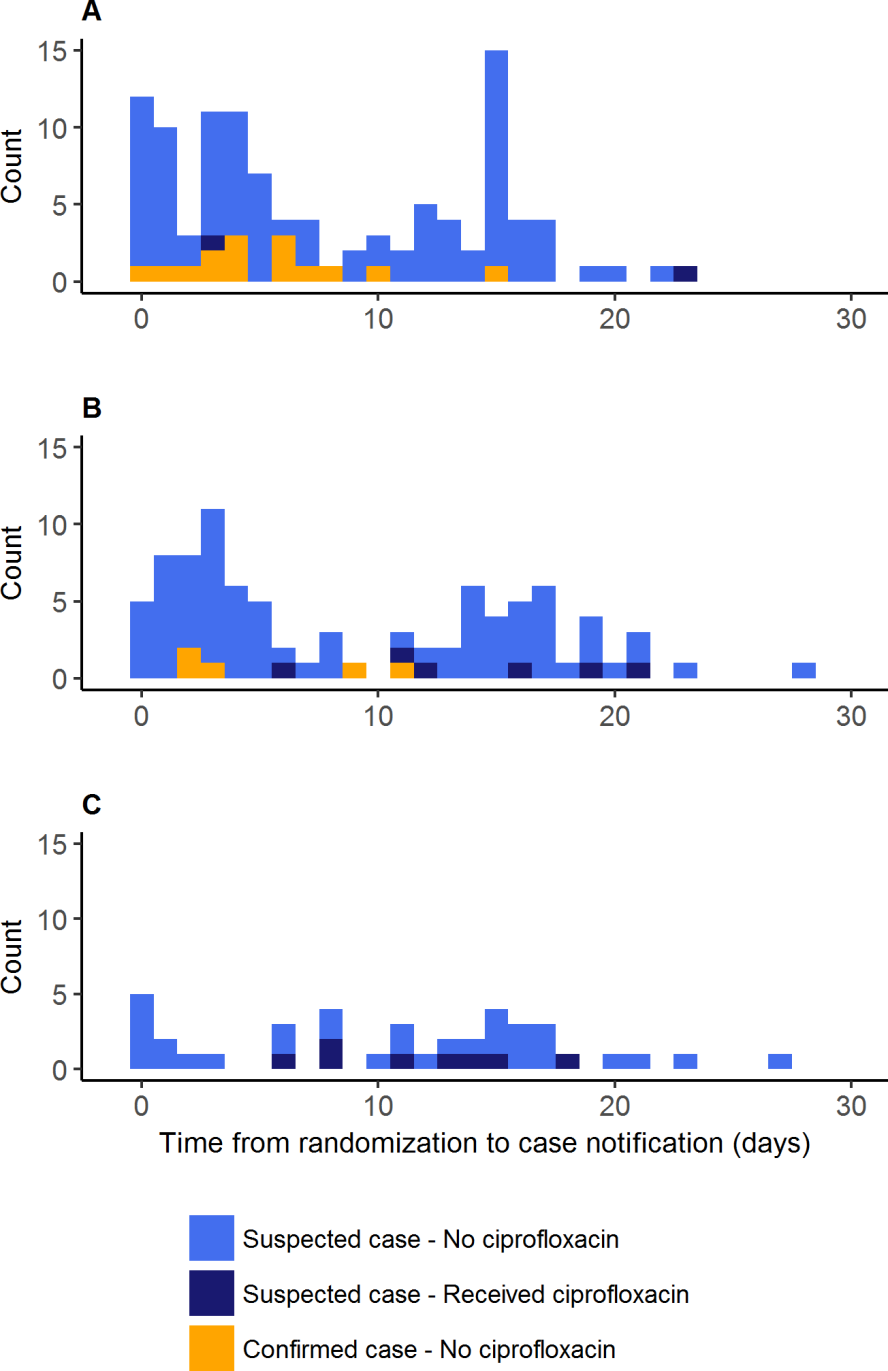
Cases of meningitis over time after inclusion, Madarounfa District, Niger, 2017. (A) Control arm. (B) Household prophylaxis arm. (C) Village-wide prophylaxis arm.

ARs were consistently lower in all age groups in the village-wide prophylaxis arm compared to the control arm (Table 3), but confidence intervals are wide and overlapping as the study was not powered to detect age-specific effects. Similarly, the AR ratios comparing village-wide prophylaxis to control were 0.34 among females and 0.49 among males. Female cases were older than male cases (mean age 20 years versus 14 years, *p* < 0.001). Fig 3 presents the timing of the case notification in each of the 3 arms for case patients whose data on treatment with ciprofloxacin was available (237/248 suspected cases).

**Table 3:** Attack rates by age, sex, and case confirmation status.

Treatment arm and group	Cases post-inclusion	Population at risk	Attack rate per 100,000 persons (95% CI)*
**Sex**			
Male			
Control	47	12,465	377.1 (228.7‒621.7)
Household prophylaxis	40	11,469	348.8 (193.6‒628.2)
Village-wide prophylaxis	21	10,882	193.0 (100.6‒370.1)
Female			
Control	68	13,028	521.9 (276.7‒984.3)
Household prophylaxis	51	12,135	420.3 (244.2‒723.5)
Village-wide prophylaxis	21	11,280	186.2 (85.8‒403.8)
**Age in years**			
Under 5			
Control	18	5,984	300.8 (157.4‒574.9)
Household prophylaxis	19	5,765	329.6 (149.2‒728.3)
Village-wide prophylaxis	14	5,358	261.3 (124.1‒550.1)
5–14			
Control	28	8,179	342.3 (181.0‒647.6)
Household prophylaxis	23	7,532	305.4 (177.9‒524.2)
Village-wide prophylaxis	7	7,026	99.6 (41.1‒241.8)
15–29			
Control	49	5,570	879.7 (438.1‒1767)
Household prophylaxis	30	4,981	602.3 (293.3‒1237)
Village-wide prophylaxis	15	4,635	323.6 (126.0‒831.3)
30 and above			
Control	20	5,760	347.2 (173.5‒694.9)
Household prophylaxis	19	5,326	356.7 (171.7‒741.4)
Village-wide prophylaxis	6	5,143	116.7 (45.4‒299.6)
**Confirmed NmC**			
Control	16	25,507	—
Household prophylaxis	5	23,618	—
Village-wide prophylaxis	0	22,176	—

NmC, *N*. *meningitidis* serogroup C.

Regarding the individual-level protective effectiveness of having taken ciprofloxacin, when comparing all persons in the study area who received ciprofloxacin (in either prophylaxis arm) to all persons who did not receive ciprofloxacin, the crude AR ratio was 0.18 (95% CI 0.10‒0.33), corresponding to a protective effectiveness of 82% (95% CI 67%‒90%, *p* < 0.001). Individual-level covariate information was not available for non-cases, so it was not possible to calculate adjusted estimates.

Of the 297 overall suspected cases in the study area (including cases that triggered village inclusion), 74 had CSF samples analyzed by PCR: 28 (38%) were positive for NmC and 2 (3%) were positive for *Streptococcus pneumoniae*. The rest were negative. Among the 248 post-inclusion cases, 52 samples were analyzed by PCR, of which 21 (40%) were positive for NmC and 31 (60%) were negative. In the control arm, 16/28 samples (57%) tested positive for NmC; in the household prophylaxis arm, 5/16 samples (31%) tested positive for NmC; and in the village-wide prophylaxis arm, 0/8 samples tested positive for NmC. The occurrence of 0 cases in the village-wide prophylaxis arm was interpreted to be due to a low number of cases tested. A point estimate for effectiveness against confirmed NmC of 100% is therefore likely not valid, and thus neither would statistical inference based on the AR for confirmed meningitis cases be valid. Among the 49 cases that triggered village inclusion, 22 had CSF samples analyzed by PCR, and 7 were positive for NmC: 3 in the control arm, 3 in the household prophylaxis arm, and 1 in the village-wide prophylaxis arm; 2 were positive for *S*. *pneumoniae*: 1 in the control arm and 1 in the household prophylaxis arm.

No serious adverse events were reported.

### Results of the antibiotic resistance sub-study

Prevalence of fecal carriage of ciprofloxacin-resistant and ESBL-producing Enterobacteriaceae was high at baseline in the village-wide prophylaxis and control arms (Table 4). There was no difference in the change of prevalence over time between the arms. One hundred samples were sent for external quality control, of which 99 grew and 98 were identified as *Escherichia coli* by mass spectrometry (MALDI-TOF). Results of antimicrobial resistance testing in the reference laboratory were concordant with results obtained in the field for 98 of the 99 samples tested.

**Table 4:** Prevalence of fecal carriage of resistant Enterobacteriaceae.

Outcome	Control, *n/N* (%)	Village-wide prophylaxis, *n/N* (%)	Difference in differences from day 0 (95% CI), *p-*value
**CiproR**			
Day 0	189/198 (95.5%)	175/185 (94.6%)	
Day 7	179/192 (93.2%)	176/181 (97.2%)	4.9 (−1.0 to 10.8), *p* = 0.11
Day 28	183/193 (94.8%)	178/180 (98.9%)	5.0 (−0.4 to 10.3), *p* = 0.07
**ESBL-producing bacteria**			
Day 0	181/198 (91.4%)	173/185 (93.5%)	
Day 7	167/192 (87.0%)	168/181 (92.8%)	3.8 (−3.8 to 11.3), *p* = 0.33
Day 28	179/193 (92.7%)	167/180 (92.8%)	−2.0 (−9.0 to 5.0), *p* = 0.58
**CiproR + ESBL-producing bacteria**			
Day 0	130/196 (66.3%)	122/184 (66.3%)	
Day 7	139/192 (72.4%)	150/181 (82.9%)	10.7 (−0.1 to 22.3), *p* = 0.07
Day 28	139/193 (72.0%)	138/180 (76.7%)	4.7 (−8.1 to 17.5), *p* = 0.47

CiproR, resistance to ciprofloxacin; ESBL, extended-spectrum beta-lactamase.

## Discussion

In the setting of an NmC outbreak in a rural district of the African meningitis belt, village-wide prophylaxis with single-dose oral ciprofloxacin within 72 hours of the notification of the first suspected case from a village was associated with a 60% reduction in the overall meningitis AR. These results suggest that chemoprophylaxis is a promising epidemic response strategy, particularly given the shortage of available vaccines in the foreseeable future and the delays and logistical burden inherent with reactive vaccination campaigns. The effect was seen across all age groups. Fig 3 illustrates that the reduction in cases seen as a result of village-wide prophylaxis came in the first few days, consistent with rapid clearance of meningococcal carriage from the nasopharynx, further underscoring the importance of rapid responses during highly localized meningitis outbreaks.

This epidemic occurred late in the epidemic season, and was relatively small by historical standards. Its short duration meant that our predefined strategy of setting a target sample size after 4 weeks of inclusions was not possible. At least 2 assumptions retained for our original sample size contingency table were faulty, with the actual ICC much lower than assumed, and village size somewhat larger. This is not surprising given the novelty of this type of study and lack of previous data to inform hypotheses.

The strategy of providing household contacts with ciprofloxacin prophylaxis was not associated with decreased overall AR. Secondary analysis showed an individual-level protective effectiveness of taking ciprofloxacin of 82%, similar to estimates from high-resource settings. Taken together, these 2 findings suggest that offering prophylaxis to household contacts is likely beneficial for an individual, since household contacts are at higher risk of meningitis than the general population during meningitis belt outbreaks [32], but that it might not be effective as an outbreak control strategy.

Our estimates of AR ratios were adjusted for the timing of the first rains, which likely had an effect on the dynamics of the epidemic [2]. And although 47 of 49 villages included in this study were eventually vaccinated with an AC polysaccharide vaccine, the timing of vaccination (3–4 weeks after the study started, depending on the village), combined with the fact that the protective effect is not seen until 7–14 days post-vaccination [33], makes it unlikely that the vaccination campaign played a major role in ending the epidemic.

Vaccination remains the only recommended preventive activity during meningitis outbreaks, and has benefits over antibiotic prophylaxis, including conferring protection during subsequent years. On the other hand, oral ciprofloxacin is cheaper than currently available vaccines, is easier to administer, is not stored in a cold chain, and does not involve injections and their subsequent waste management. Meningitis belt countries are currently encouraged to have vaccine stockpiles in-country, but given problems with supply, cost, and storage, this is simply not realistic for most countries. The long shelf life and relatively low price of ciprofloxacin mean that it could conceivably be stockpiled in-country for rapid deployment.

The high levels of fecal carriage of ciprofloxacin-resistant and ESBL-producing Enterobacteriaceae are concerning. Previous data from the study area showed carriage of ESBL-producing Enterobacteriaceae in 31% of malnourished children on admission to hospital [34]. To our knowledge, the highest prevalence of ESBL carriage in healthy individuals in sub-Saharan Africa is 59%, reported in Central African Republic [35]. While we did not show that village-wide distributions of ciprofloxacin led to any significant increase in the prevalence of carriage of resistant Enterobacteriaceae, our study was underpowered to show any changes given the higher-than-expected baseline prevalence. Future evaluations of large-scale ciprofloxacin prophylaxis should continue investigating antimicrobial resistance among respiratory and gastrointestinal bacteria in other settings across the meningitis belt, and routine surveillance of resistance patterns, including among strains isolated from asymptomatic nasopharyngeal carriers, should be reinforced in settings where the strategy has been used.

No CSF samples from the epidemic were inoculated in trans-isolate medium, so meningococcal culture and antibiotic susceptibility testing were not performed in case patients, but we are reassured that ciprofloxacin is not used for the treatment of meningococcal meningitis. Meningococcal resistance to ciprofloxacin has not been commonly reported [36], but clusters of resistant strains, both in case patients and among nasopharyngeal carriage samples in asymptomatic individuals, have been reported in India and China [37,38]. This underscores the importance of continued investigation of antimicrobial resistance patterns among meningococci during future research on the use of large-scale prophylaxis.

Another limitation of this study was the small number of PCR-confirmed cases, though this is common in the meningitis belt, where during the period 2011–2013, only 7% of all suspected cases were eventually confirmed for any pathogen by PCR [39]. We also note that the proportion of suspected case patients who benefitted from confirmatory testing was comparable to that in previous epidemics in Niger [8]. Nonetheless, the patterns seen among confirmed cases reinforce the primary results, and sensitivity analyses looking at ARs post-intervention (not simply post-inclusion, which was the prespecified primary analysis) did not show differences in the primary outcome. The lack of specificity of the standard case definitions used in the trial likely means that some suspected cases did not actually have meningitis, but we expect these cases to have been equally distributed among study arms. We did not include the use of a placebo for the control group, so our study could not be masked. As such, we cannot exclude the possibility that some of the effect seen may have been due to a perceived benefit by persons receiving ciprofloxacin, leading them to be less likely to seek care. However, we believe that cluster randomization of discrete villages provided a realistic view of what would happen if a village-wide prophylaxis strategy were adopted. We do note that there was some spillover of treatment groups (2 suspected cases from the control arm reported having taken ciprofloxacin). This is an inherent difficulty of conducting this type of research in an epidemic, particularly with a well-known and widely available drug. The use of passive, facility-based surveillance could have introduced a reporting bias, but this bias should not have differed among study arms, as randomization occurred at the village level.

It will be important to evaluate the use of large-scale prophylaxis in an urban area, where high population density and different patterns of human movement may lead to different effectiveness. Several aspects of organizing ciprofloxacin distributions in a large urban center could prove challenging, such as crafting social mobilization messaging, delineating areas to receive distributions, and ensuring use of quality drugs, especially since ciprofloxacin of unknown quality is commonly available in markets without prescription. Furthermore, giving a large group of people ciprofloxacin at the same time would likely be key to success in an urban area, but even small neighborhoods of a large city would be larger than the villages included in this study. The duration of the impact of distributions of ciprofloxacin should also be investigated if they occur early in the meningitis season, especially since the strategy could be used as a stopgap measure until reactive vaccination begins. Evaluation earlier in the season may also provide information about the length of protection from a single dose of ciprofloxacin, which may be limited. Indeed, if multiple doses of ciprofloxacin were necessary to provide protection over the course of a longer epidemic, this could impact the development of antimicrobial resistance. There is no plausible biological reason to believe that ciprofloxacin prophylaxis would not be as effective in outbreaks of other meningococcal serogroups. Given that there is no vaccine available against *N*. *meningitidis* serogroup X, prophylaxis seems to be the only potential response strategy available against an outbreak of this serogroup.

The persistence of seasonal meningococcal meningitis epidemics in the African meningitis belt can ultimately be broken only with an effective and affordable conjugate vaccine covering all serogroups of epidemic potential. A pentavalent vaccine for the African market against serogroups A, C, W, Y, and X is currently under development but will not be available for several years. In the interim, large-scale prophylaxis with ciprofloxacin as an epidemic response could be a valuable tool, and should continue to be evaluated.

## Supporting information

S1 CONSORT Checklist (DOCX)

S1 Fig Ciprofloxacin coverage versus attack rate. (TIF)

S1 Protocol Cluster-randomized trial to evaluate the impact of ciprofloxacin for contact cases of meningococcal meningitis as an epidemic response. (DOCX)

S1 Table Ciprofloxacin dosing. (DOCX)

S1 Timeline Protocol/statistical analysis plan timeline. (DOCX)

## Abbreviations

AR: attack rate
CSF: cerebrospinal fluid
ESBL: extended-spectrum beta-lactamase
HA: health area
HC: health center
ICC: intra-cluster correlation coefficient
NmA: *Neisseria meningitidis* serogroup A
NmC: *Neisseria meningitidis* serogroup C
WHO: World Health Organization
