# Supplementary figures and images for: Single-dose oral ciprofloxacin prophylaxis as a response to a meningococcal meningitis epidemic in the African meningitis belt: A 3-arm, open-label, cluster-randomized trial
Source: PLoS Med. 2018 Jun 26;15(6):e1002593. doi: 10.1371/journal.pmed.1002593 (PMC6019097; doi:10.1371/journal.pmed.1002593)

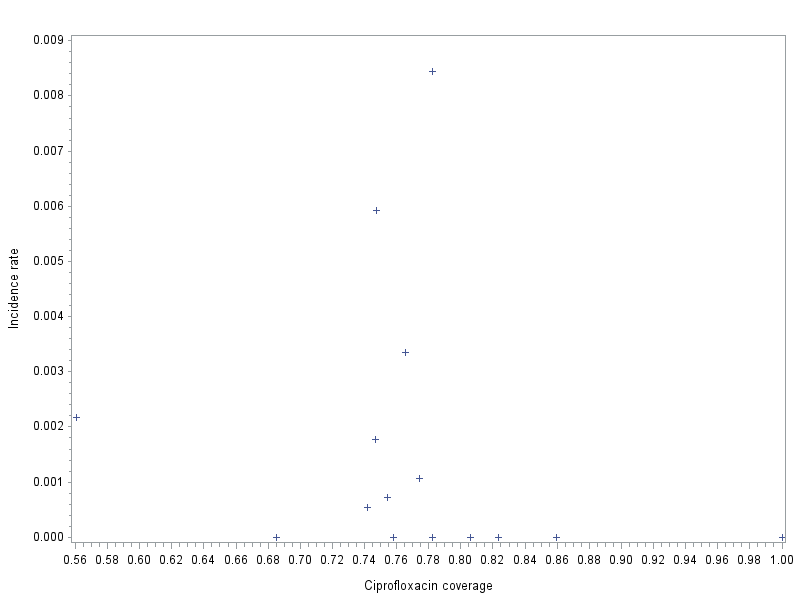

Supplement: S1 Fig — (TIF) [file pmed.1002593.s002.tif]
