# Supplementary material for: Single-dose oral ciprofloxacin prophylaxis as a response to a meningococcal meningitis epidemic in the African meningitis belt: A 3-arm, open-label, cluster-randomized trial
Source: PLoS Med. 2018 Jun 26;15(6):e1002593. doi: 10.1371/journal.pmed.1002593 (PMC6019097; doi:10.1371/journal.pmed.1002593)
Supplement: S1 Protocol — (DOCX) [file pmed.1002593.s003.docx]

mc andgular

Cluster-randomized trial to evaluate the impact of ciprofloxacin for contacts of cases of meningococcal meningitis as an epidemic response

Study Protocol

Version 1.5

April 18, 2016


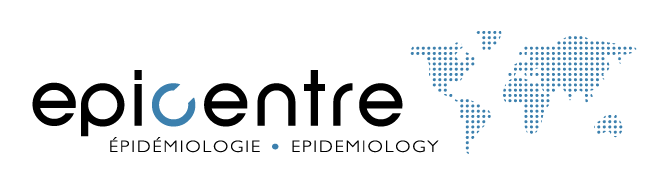


. *Téléphone :* 00 33 (0)1 40 21 28 48

*Fax :* 00 33 (0)1 40 21 28 03

*E-Mail :* Epimail@Epicentre.Msf.Org

*Web :* http://www.Epicentre.Msf.Org

Association loi 1901


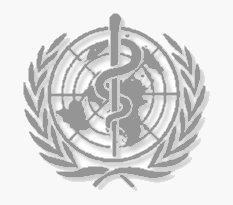


Centre Collaborateur de l’OMS
pour la Recherche en Epidémiologie
et la Réponse aux Maladies Emergentes

| **Title** | Cluster-randomized trial of ciprofloxacin for contacts of cases of meningococcal meningitis as an epidemic response |
| --- | --- |
| **Study Site** | A Health District experiencing a meningitis epidemic in Niger |
| **Primary Sponsor** | Epicentre  8 rue Saint Sabin  Paris 75011, France |
| **Secondary Sponsor** | Médecins Sans Frontières-Operational Center Geneva  78 rue de Lausanne  CP 116  1211 Geneva 21, Switzerland |
| **Sponsor Study Director** | Rebecca F. Grais, PhD  Epicentre  8 rue Saint Sabin  Paris 75011, France  Tel : + 33 1 4021 5475  Email : rebecca.grais@epicentre.msf.org |
| **Sponsor Principal Investigator** | Matthew Coldiron, MD, MPH  Epicentre  8 rue Saint Sabin  Paris 75011, France  Tel : + 33 1 4021 5498  Email : matthew.coldiron@epicentre.msf.org |
| **Primary Co-investigator** | Gabriel Alcoba, MD, MPH  Médecins Sans Frontières – Operational Center Geneva  78 rue de Lausanne  CP 116  1211 Geneva 21, Switzerland |
| **Co-Investigators** | Céline Langendorf, PhD, MPH, Epicentre Paris  Anne-Laure Page, PhD, Epicentre Paris  Ali Djibo, MD, Niamey National Hospital, Niger  Eric Adehossi, MD, Niamey National Hospital, Niger  Dorian Job, MD, MSF-OCG, Geneva  Iza Cignelecki, MD, MPH, MSF-OCG, Geneva  Michel Quere, MD, MSF-OCG, Geneva |
| **Field Principal Investigator** | Oumar Touré, MD  Boite Postale 419  Maradi, Niger  oumar.toure@epicentre.msf.org |

| **Participating Laboratories** | Epicentre Maradi Laboratory  Boite Postal 419  Maradi, Niger  Email : nathan.sayinzoga-makombe@epicentre.msf.org |
| --- | --- |
| **Protocol Version** | Version 1.5 (Issue date: April 18, 2016) |

**INVESTIGATOR SIGNATURE PAGE**

We, the undersigned, have read and understood this protocol. We hereby agree to conduct the study in accordance with this protocol and to comply with all requirements regarding the obligations of investigators, the ethical principles that have their origin in the Declaration of Helsinki and all other pertinent requirements of the ICH Harmonized Tripartite Guideline and the local public health authorities.

We agree to comply with all relevant SOPs required for the conduct of this study. We further agree to ensure that all persons assisting in the conduct of this study are informed regarding their obligations.

____________________________________ ____________________

Sponsor Study Director Date

Dr. Rebecca Freeman Grais

Epicentre, 8 rue Saint Sabin, 75011 Paris France

Email: rebecca.grais@epicentre.msf.org

Tel: +33 (0)1 4021 5475

____________________________________ ____________________

Secondary Sponsor’s Representative: Date

Gabriel Alcoba

Médecins Sans Frontières-Operational Center

Geneva

Email: gabriel.alcoba@geneva.msf.org

____________________________________ ____________________

Primary Sponsor’s Representative: Date

Matthew Coldiron

Epicentre, 8 rue Saint Sabin, 75011 Paris France

Email: matthew.coldiron@epicentre.msf.org

Tel: +33 (0)1 4021 5506

**TRIAL REGISTRATION DATA AND SUMMARY**

**Primary Registry and Trial Identifying Number:** To be assigned

**Date of Registration in Primary Registry:** NA

**Secondary Identifying Numbers:** None

**Sources of Monetary and Material Support**

Médecins Sans Frontières-Operational Center Geneva will provide funding for the trial.

**Primary Sponsor**

Epicentre takes responsibility for initiating, registering and conducting the trial, and as such, will be involved in the study design; collection, management and analysis, and interpretation of data; and writing of the report. Epicentre takes responsibility for ensuring the trial is properly monitored and results are made available.

Primary sponsor contact: Dr. Rebecca Freeman Grais, Director, Department of Research and Population Health, Epicentre (rebecca.grais@epicentre.msf.org; Tel: +33 (0)1 4021 5475; Address: Epicentre, 8 rue Saint Sabin, 75011 Paris France)

**Secondary Sponsor**

Médecins Sans Frontières- Operational Center Geneva has agreed with the primary sponsor to act as the secondary sponsor and the primary sponsor’s legal representative in relation to the trial site and provide funding for the trial. The secondary sponsor will be involved in the study design, interpretation of data and writing of the report.

Secondary sponsor contact: Dr. Gabriel Alcoba (Tropical Medicine Advisor, Médecins Sans Frontières-Operational Center Geneva; Address: 78 rue de Lausanne, CP 116, 1211 Geneva 21, Switzerland; Email: Gabriel.alcoba@geneva.msf.org)

**Public Title**

Cluster-randomized trial of ciprofloxacin for contacts of cases of meningococcal meningitis as an epidemic response

**Scientific Title**

Cluster-randomized trial of ciprofloxacin for contacts of cases of meningococcal meningitis as an epidemic response

**Health Condition(s) or Problem(s) Studied**

Meningococcal meningitis during an epidemic

**Interventions**

Oral ciprofloxacin for household or community contacts of meningitis cases during an epidemic. Community sensitization messages about signs and symptoms of meningitis.

**Key Inclusion and Exclusion Criteria**

The study will be performed in villages of a single health district where at least 2 Health Areas have passed epidemic thresholds of 10 cases per 100 000 residents per week. All villages in the affected health areas will be eligible for randomization, and will be included if the village chief provides written informed consent.

In the arms where ciprofloxacin is administered, the individuals who show signs or symptoms of meningitis at the time of ciprofloxacin distribution will be excluded. These individuals will be immediately transported to the nearest health center for diagnosis and treatment following national protocols. Individuals with known allergy to fluoroquinolones will not be eligible to receive ciprofloxacin.

**Study Type**

Interventional, cluster-randomized, parallel three-arm trial to assess the impact of oral ciprofloxacin for household and community contacts of meningitis cases on the incidence of meningitis during an epidemic. The trial contains a nested sub-study (“resistance study”) to assess the effect of a single dose of ciprofloxacin on the prevalence of fluoroquinolone-resistant enterobacteriaceae in the study area.

**Study design**

The study is designed as a cluster-randomized trial with three parallel arms in the setting of a meningitis epidemic to assess the impact of chemoprophylaxis with single-dose ciprofloxacin on the incidence of meningitis in the study area.

The study will be implemented in a health district where a meningitis outbreak is occurring, and where Médecins Sans Frontières is providing assistance to the Ministry of Public Health in its outbreak response. In order to launch the study protocol, at least two Health Areas (HA) of the Health District (HD) will have met the weekly epidemic threshold of 10 cases per 100 000 per week, or of 5 cases per week if the population of the HA is less than 30 000 people, following the most recent WHO recommendations for meningitis surveillance.

All villages in the HAs which have crossed the epidemic threshold and are included in the study area will be randomized in a 1:1:1 ratio to receive standard care, household-level prophylaxis, or community-wide prophylaxis.

Once a HA has been included in the study, a case-based surveillance system will be put in place, or reinforced if one is currently in place in the study area. Suspected cases of meningitis that present to health posts will be referred to the nearest health center. In each health center, the diagnosis and treatment of meningitis will follow national protocols.

In the standard care arm, after the first case has been notified from a village, a study nurse will visit the village with a local community member. The study nurse will lead an informational session for members of the community regarding the signs and symptoms of meningitis, and the urgency of presenting to the nearest health center as soon as possible if any of those signs or symptoms arrive. In the household-level prophylaxis arm, each time a case is reported from a village that has been randomized to this arm, a study nurse will visit the household of the notified case within 24 hours. The study nurse will offer ciprofloxacin to all persons currently living in the same household compound and present at the time of the visit. In the community-level prophylaxis arm, after the first case is reported from a village that has been randomized to this arm, a member of study staff will visit the village within 24 hours. During this visit, arrangements will be made for a community-wide distribution of ciprofloxacin, preferably within 72 hours of the initial case presentation at the health center / district hospital. A series of informational meetings will be organized before the distribution.

A nested substudy to assess the effects of ciprofloxacin prophylaxis on the prevalence of ciprofloxacin-resistant enterobacteriaceae will be carried out among 400 persons (200 in the standard care arm and 200 in the community-wide prophylaxis arm). Participants will provide a series of 3 stool samples for culture, speciation, and antibiotic resistance testing.

**Tentative Date of First Enrollment**

2016 epidemic meningitis season

**Sample Size**

The sample size needed to show a difference in the reduction of the meningitis attack rate will be predicated on multiple factors, some of which can be reliably estimated (risk of developing meningitis among household contacts of cases), and others which cannot be reliably estimated, or which will depend on the study area. The overall size of the epidemic (as measured by cumulative incidence) is not reliably predictable. Average household size and average village size also vary significantly by region of Niger.

Because of these uncertainties, the overall target sample size necessary will be set after four weeks of accumulated study data. This period is sufficiently long to allow for a description of the demographic co-factors in the study area. A table presenting different scenarios shows the high variability given these co-factors, but the most likely combinations of scenarios present sample sizes that are logistically feasible.

**Study objectives**

Primary objective

Compare the incidence of meningitis among villages receiving standard care, household ciprofloxacin prophylaxis and village-wide prophylaxis.

Secondary objectives:

- Compare the incidence of meningitis by sex in the three different intervention areas.
- Compare the incidence of meningitis by age in the three different intervention areas.
- Estimate the individual efficacy of oral ciprofloxacin for the prevention of meningitis.
- Compare the prevalence of enterobacteriaceae resistant to ciprofloxacin before and after distributions of ciprofloxacin in communities receiving distributions versus in communities not receiving distributions.

**ABBREVIATIONS**

CIOMS Council for International Organizations of Medical Sciences

CRF Case report form

CSF Cerebrospinal fluid

EMA European Medicines Agency

FDA United States Food and Drug Administration

GCP Good clinical practice

HA Health area

HD Health district

ICH International Conference on Harmonization

IRB Institutional Review Board

MIC Minimum inhibitory concentration

MPH Ministry of Public Health

MSF Médecins Sans Frontières

MSF-OCG Médecins Sans Frontières – Geneva Operational Center

Nm *Neisseria meningitidis*

NmA *Neisseria meningitidis* serogroup A

NmC *Neisseria meningitidis* serogroup C

NmW *Neisseria meningitidis* serogroup W

NmX *Neisseria meningitidis* serogroup X

NmY *Neisseria meningitidis* serogroup Y

SAE Serious adverse event

SOP Standard operating procedure

WHO World Health Organization

**TABLE OF CONTENTS**

[INVESTIGATOR SIGNATURE PAGE 4](#_Toc444189025)

[TRIAL REGISTRATION DATA AND SUMMARY 5](#_Toc444189026)

[ABBREVIATIONS 9](#_Toc444189027)

[TABLE OF CONTENTS 10](#_Toc444189028)

[BACKGROUND AND RATIONALE 13](#_Toc444189029)

[**Meningococcal meningitis** 13](#_Toc444189030)

[**Nasopharyngeal carriage, transmission dynamics and propagation of epidemics** 13](#_Toc444189031)

[**MenAfriVac** 14](#_Toc444189032)

[**Emergence of NmC** 15](#_Toc444189033)

[**Prevention of meningococcal meningitis** 15](#_Toc444189034)

[**Study rationale** 16](#_Toc444189035)

[STUDY OBJECTIVES 17](#_Toc444189036)

[METHODOLOGY 18](#_Toc444189037)

[**Overall study design and study launch criteria** 18](#_Toc444189038)

[**Study setting** 19](#_Toc444189039)

[**Ciprofloxacin** 19](#_Toc444189040)

[**Randomization procedures** 20](#_Toc444189041)

[**Meningitis surveillance and incidence calculations** 21](#_Toc444189042)

[**Target population** 22](#_Toc444189043)

[**Study sites** 22](#_Toc444189044)

[**Study procedures** 22](#_Toc444189045)

[*Interventions* 22](#_Toc444189046)

[*Dosing and administration of ciprofloxacin* 23](#_Toc444189047)

[**Standard care for meningitis** 24](#_Toc444189048)

[**Sample size** 24](#_Toc444189049)

[**Resistance sub-study** 25](#_Toc444189050)

[**Data analysis** 27](#_Toc444189051)

[**Data collection, management and quality assurance** 27](#_Toc444189052)

[**Timeline** 28](#_Toc444189053)

[SAFETY 29](#_Toc444189054)

[**Documenting serious adverse events** 29](#_Toc444189055)

[**Assessment of causality** 30](#_Toc444189056)

[MONITORING 31](#_Toc444189057)

[ETHICAL CONSIDERATIONS 32](#_Toc444189058)

[**Ethical considerations particular to cluster-randomized trials** 32](#_Toc444189059)

[**Summary of known and potential risks, identification of vulnerable populations** 33](#_Toc444189060)

[**Risk minimization and benefits** 33](#_Toc444189061)

[**Definition of research subject and identification of gatekeepers** 34](#_Toc444189062)

[**Informed consent** 34](#_Toc444189063)

[**Confidentiality** 36](#_Toc444189064)

[**Reimbursement** 36](#_Toc444189065)

[**Storage of specimens** 36](#_Toc444189066)

[**Institutional Review Board approval** 37](#_Toc444189067)

[**Declaration of conflict of interests** 37](#_Toc444189068)

[STUDY ADMINISTRATION 38](#_Toc444189069)

[**Protocol amendments** 38](#_Toc444189070)

[**Protocol deviations and violations** 38](#_Toc444189071)

[**Ancillary care and insurance** 38](#_Toc444189072)

[**Data storage and archival** 39](#_Toc444189073)

[**Dissemination and authorship policy** 39](#_Toc444189074)

[**Data sharing policy** 39](#_Toc444189075)

[STUDY MANAGEMENT 40](#_Toc444189076)

[**Study sponsors** 40](#_Toc444189077)

[**Scientific Committee** 40](#_Toc444189078)

[**Human resources** 40](#_Toc444189079)

[**Training** 41](#_Toc444189080)

[REFERENCES 44](#_Toc444189081)

**APPENDICES**

Appendix A. Village leader informational notice and written permission document

Appendix B. Informational notice for persons eligible to receive ciprofloxacin

Appendix C. Informational notice and consent document for resistance sub-study

Appendix D. Terms of Reference and Membership, Scientific Committee

Appendix E. Declaration of Helsinki

**BACKGROUND AND RATIONALE**

**Meningococcal meningitis**

Epidemics of meningococcal meningitis have been described in Africa since 1840 (Greenwood, 1999), and large-scale, cyclic epidemics have been described throughout the Sahel during the last century (Lapeyssonie, 1963). These epidemics have given rise to the term “African meningitis belt”, which stretches from Senegal to Sudan and Ethiopia (Molesworth, 2002).

Historically, the largest epidemics of meningococcal meningitis in the meningitis belt were caused by Neisseria meningitidis serogroup A (NmA). The 1996 epidemic in Nigeria was associated with over 100 000 cases and over 10 000 deaths (Mohammed, 2000). Nonetheless, epidemics caused by serogroups W (NmW) and X (NmX) have been described (Nathan, 2007; Boisier, 2007). Other serogroups, such as NmB, NmC and NmY have been associated with smaller-scale epidemics in Europe and the Americas. The virulence of the different strains is not necessarily associated with one serogroup or another; rather, different proteins and polysaccharides expressed on the capsular membranes are highly associated with virulence (Stephens, 2007).

**Nasopharyngeal carriage, transmission dynamics and propagation of epidemics**

The epidemiology of meningococcal meningitis has been well described, but understanding of the mechanisms of infection, transmission and propagation of epidemics is less well understood. Nasopharyngeal carriage of *Neisseria* and local meteorological conditions play important roles in both the natural history of the disease in individuals and also of epidemics. Crowded conditions and interhuman transmission may also play roles.

Nasopharyngeal carriage of meningococci is common in at-risk populations, with a prevalence that can very between 3 and 30% in different contexts and different times of the year (Trotter, 2007). The rate of carriage in a community is generally higher during epidemic periods than during non-epidemic periods (Stephens, 2007). Nonetheless, nasopharyngeal carriage of meningococci alone is not enough to lead to invasive forms of meningococcal disease. Of note, nasopharyngeal carriage of meningococci has been shown to be higher among members of the immediate family of cases of meningococcal meningitis (Greenwood, 1978).

In the meningitis belt, the large epidemics occur during the dry season, usually between January and May. This time of year is very dry, with low ambient humidity, relatively lower temperatures, and is often extremely dusty (Sultan, 2005). These conditions can have an effect on the integrity of the nasopharyngeal mucosae, and in a person who has been colonized; this can lead to invasive disease.

Another important element is acquired immunity. After infection, or after vaccination with a polysaccharide vaccine for a certain serogroup, a short period of immunity is gained: 2-3 years for NmA, and perhaps even less for other serogroups (McIntyre, 2012). Vaccination with meningococcal conjugate vaccines provokes much longer-lasting immunity, and also blocks nasopharyngeal carriage (Kristiansen, 2013). Polysaccharide vaccination, on the other hand, does not prevent nasopharyngeal carriage (Dellicour, 2007).

Several explanations exist to bring these various factors together, but one of the most convincing is that described by Mueller and Gessner. They describe a two-step series of changes. First, the change in humidity between the rainy and dry seasons causes a change between endemicity and hyperendemicity, possibly due to increasing rates of nasopharyngeal carriage. But a second change is necessary to provoke large-scale epidemics, such as the circulation of a new strain of meningococcus or a concomitant upper respiratory illness in a community (Mueller, 2010).

In this explicative model, most epidemics remain localized – incidence is extremely high in a very local area, with only occasional large-scale epidemics. Evidence supporting this hypothesis, at least for NmA, has recently been published (Koutangni, 2015). The authors show that the transition between the rainy season and the dry season is associated with a change in the case-carriage ratio, but that the transition between the hyperendemic phase and epidemics is marked by higher transmission.

**MenAfriVac**

The introduction of a conjugate vaccine against NmA (MenAfriVac) in 2010 has had a major impact on the incidence of meningitis in the African meningitis belt (Figure 1). Immunity may last for even longer than 10 years, and carriage of NmA has been virtually eliminated (Kristiansen, 2013). A study in Chad, partially vaccinated in 2011, showed a 94% reduction in meningitis in 2012 in vaccinated zones compared to non-vaccinated zones (Daugla, 2014). Figure 1 shows the number of notified meningitis cases in the meningitis belt between 1994 and 2014; a substantial reduction of cases occurred after the introduction of MenAfriVac in 2010.

**Figure 1:** Meningitis cases notified in the meningitis belt, 1994-2014


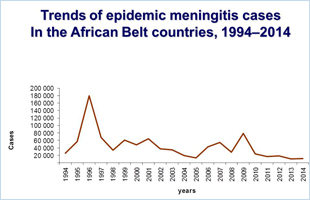


*Source : WHO*

**Emergence of NmC**

In the relative calm post-MenAfriVac introduction, small-scale epidemics caused by NmC were notified in northwest Nigeria in 2013 and 2014. In Sokoto State in 2013, a total of 856 cases were notified (attack rate 673 per 100 000 persons, case fatality rate 6.8%). In Kebbi state in 2014, a total of 333 cases were notified (attack rate 165 per 100 000, case fatality rate 10.5%) The strain of NmC responsible for these epidemics has been fully described; it is a novel strain (Funk 2014). These epidemics were the first NmC epidemics described in the meningitis belt since a 1979 epidemic in Burkina Faso (Broome, 1983) and a 1975 epidemic in Nigeria (Whittle, 1975).

In 2015, Sokoto and Kebbi states, which border Niger, reported over 6000 cases of meningitis beginning in January. The epidemic spread to bordering regions of Niger, and then to Niger’s capital, Niamey. In Niger, over 8000 cases were reported.

The response to the epidemic in 2015 was complicated by an overall lack of available vaccine (polysaccharide or conjugate) containing antigens against NmC. The availability of NmC-containing vaccines is expected to be limited for the 2016 meningitis season as well, with only 1.4 million doses guaranteed available in January, with a maximum of 5 million doses available by April. Given the rapid expansion of NmC in Nigeria and Niger, and its appearance in routine case-based surveillance data in Ghana, Mali, and Burkina Faso at the end of the 2015 season, this is a particularly worrisome situation, as the risk of large-scale epidemics due to NmC is judged to be extremely high (WHO, 2015). Supply of NmC-containing vaccines is expected to be limited for the foreseeable future, as development of a multivalent conjugate vaccine by the manufacturer of MenAfriVac (targeted for release between 2020-2022 pending results of studies and licensure) has dampened interest from other vaccine producers in the interim.

**Prevention of meningococcal meningitis**

The prevention of meningococcal meningitis is based largely on two measures: mass vaccination (either in a preventive strategy as with the roll-out of MenAfriVac, or in reactive campaigns during epidemics) and antibiotic prophylaxis. The recommendations for the use of these two strategies vary greatly depending on context.

In the African meningitis belt, the emphasis has historically been on reactive mass vaccination campaigns after the declaration of an epidemic in a given area. Unfortunately, because mass vaccination campaigns can be difficult and time-consuming to organize, and because epidemics due to NmA are often explosive and short-lived, the impact of such reactive vaccination campaigns in the meningitis belt has been called into question (Stephens, 2007; Ferrari, 2014).

On the other hand, antibiotic chemoprophylaxis was implemented in the pre-vaccine era in sub-Saharan Africa (Greenwood, 1999) and is commonly used among household contacts of cases in western countries (Stephens, 2007; Zalmanovici, 2013).

In western countries, it has been estimated that household contacts of cases of meningococcal disease have up to a 100-fold increased risk of contracting meningococcal disease (Rosenstein, 2001). Given that the epidemiology of meningococcal disease in the African meningitis belt is quite dissimilar to that of other settings in the Europe and the Americas, it is possible that the risk for household contacts is not as elevated. Indeed, results from a study in a rural district of Niger during the 2015 outbreaks showed that the attack rate for household contacts was approximately 20 times higher than that of the general community (Coldiron, unpublished 2015).

**Study rationale**

Large-scale outbreaks caused by NmC and other non-NmA serogroups are likely in the meningitis belt in 2016 and beyond. Classically, the preventive response to such outbreaks would be based on prompt identification and standard case management as well as reactive vaccination campaigns.

Given the scarcity of available vaccine for the upcoming meningitis season and in the foreseeable future, it is reasonable to pursue alternative prevention strategies, including antibiotic prophylaxis for contacts of cases. The most recent WHO recommendation on the subject for sub-Saharan Africa is that antibiotic prophylaxis for household contacts can be considered outside of epidemics, but that its routine use during epidemics was not recommended (WHO, 2014). Nonetheless, given the unexpected emergence of NmC, and the lack of available vaccine, an expert group recently convened by the WHO called for clinical trials to document the effectiveness of the use of ciprofloxacin (WHO, 2015) in epidemic contexts.

This protocol also calls for a second comparison, village-level prophylaxis. In the setting of the meningitis belt, providing household-level prophylaxis may be difficult to implement logistically, and also incur stigma against those households that do receive treatment. Given the complex relationship between nasopharyngeal carriage of meningococci, climatic conditions and human-to-human spread, it is possible that this strategy might be more effective than household-level prophylaxis at reducing the incidence of meningitis in a given area.

**STUDY OBJECTIVES**

Primary objective

Compare the incidence of meningitis among villages receiving standard care, household ciprofloxacin prophylaxis and village-wide prophylaxis.

Secondary objectives:

- Compare the incidence of meningitis by sex in the three different intervention areas.
- Compare the incidence of meningitis by age in the three different intervention areas.
- Estimate the individual efficacy of oral ciprofloxacin for the prevention of meningitis.

Substudy objective:

Compare the prevalence of enterobacteriaceae resistant to ciprofloxacin before and after distributions of ciprofloxacin in communities receiving distributions versus in communities not receiving distributions.

**METHODOLOGY**

**Overall study design and study launch criteria**

The study is designed as a cluster-randomized trial with three parallel arms in the setting of a meningitis epidemic to assess the impact of chemoprophylaxis with single-dose ciprofloxacin on the incidence of meningitis in the study area.

The study will be implemented in a health district where a meningitis outbreak is occurring, and where Médecins Sans Frontières is providing assistance to the Ministry of Public Health in its outbreak response. In order to launch the study protocol, at least two Health Areas (HA) of the Health District (HD) will have met the weekly meningitis epidemic threshold of 10 cases per 100 000 per week, or of 5 cases per week if the population of the HA is less than 30 000 people, following the most recent WHO recommendations for meningitis surveillance. Other factors (predominant causative serogroup, timing during the meningitis season, plans for reactive vaccination) will then be taken into account before seeking administrative approval in the district in question for the trial to be started.

All villages in the HAs included in the study are will be eligible for inclusion in the study. They will be randomized in a 1:1:1 ratio to receive standard care, household-level prophylaxis, or community-wide prophylaxis. Villages will receive their intervention after the notification of the first case from the village after the study has opened in a HA.

The primary aim of the study is to assess the impact of chemoprophylaxis with ciprofloxacin on the incidence of meningitis, which will be assessed until the end of the epidemic in the study area. Cases of meningitis will be identified using facility-based surveillance.

A nested substudy to describe the effects of ciprofloxacin prophylaxis on the prevalence of ciprofloxacin-resistant enterobacteriaceae will be carried out among 400 persons (200 in the standard care arm and 200 in the community-wide prophylaxis arm). Participants will provide a series of 3 stool samples for culture, speciation, and antibiotic resistance testing.

**Study setting**

Niger has historically been part of the African meningitis belt, and has known large-scale epidemics due to NmA for much of the last century. It has also seen the largest epidemic due to NmX as well as the NmC epidemic of 2015 that was described above.

The health system in Niger is a pyramidal system in line with the 1985 Lusaka agreements, based on health structures with increasing levels of service capacity: health posts (cases de santé) provide basic care and preventive services and are most often staffed by community health workers who are helped by community representatives. Health centers (centres de santé intégrés) are staffed by nurses and ensure the provision of all services not requiring hospitalization. Complications are referred to the district hospital and to the regional hospital from the district hospital. In this organization, severity signs are assessed at the level of health centers and only severe cases are referred to the district hospital.

The current protocol is designed to be implemented in an emergency setting, therefore precise details about the study location are unknown, but it will be in an area in which Médecins Sans Frontières (MSF) is supporting the Ministry of Public Health in its epidemic response.

In collaboration with the Ministry of Health, MSF has been supporting health care in Niger for many years, with a permanent presence since 2005. Projects have included medico-nutritional programs, responses to epidemics, and responses to manmade and natural catastrophes. Epicentre, the epidemiologic and research organization affiliated with MSF, has been present in Niger since 2005. In close partnership with the Ministry of Health, MSF, and other partners, Epicentre develops and conducts research aimed at responding to the medical and operational objectives of local and regional public health actors. A team of medical professionals, epidemiologists, biologists and data management specialists work in Maradi and Niamey.

**Ciprofloxacin**

Ciprofloxacin is a fluoroquinolone antibiotic that figures on the WHO list of essential medicines. Ciprofloxacin has a broad spectrum of activity, including against *E.coli,* *Campylobacter* spp*,* *Salmonella* spp, *Shigella* spp, and *P. aeruginosa*, as well as many gram-positive cocci, such as *S. pneumoniae* and *S. aureus*. It is easily absorbed and has good bio-availability. It is commonly used to treat urinary tract infections, skin and soft tissue infections, enterotoxic infections and systemic infections due to *Salmonella* and *Shigella*.

In these cases, it is administered as a several-day course, and it is generally well-tolerated. The most common side effects are gastro-intestinal, and prolonged courses have been associated with *Clostridium difficile* diarrhea.

Historically, concern has existed regarding the use of fluoroquinolones in children, as early reports suggested higher rates of chondrotoxicity and tendinopathy, including rupture of the Achilles tendon. These early associations have since been debunked (Burkhardt, 1997).

Ciprofloxacin is considered a pregnancy category C drug by the United States Food and Drug Administration, meaning that while some evidence of fetal risk has been described in animal studies, no convincing evidence exists in humans. Furthermore, this means that the benefits of treatment may warrant the use of ciprofloxacin despite the potential risks. Ciprofloxacin is the recommended antibiotic for post-exposure prophylaxis to inhalational anthrax, a disease associated with high mortality. For inhalational anthrax, the prophylaxis is for 60 days, and it is recommended for pregnant women in this setting (EMA, 2014). There is therefore a strong precedent for its use as prophylaxis for a potentially life-threatening infection among pregnant women.

Furthermore, ciprofloxacin is recommended by the WHO as an antibiotic prophylactic agent for contacts of meningitis cases in sub-Saharan Africa outside of epidemics (WHO, 2014). WHO

The greatest concern for its use in a mass administration setting would be its potential effect on antibiotic resistance, particularly among enterobacteriaceae.

Resistance of enterobacteriaceae to frequently-used antibiotics, such as trimethoprim-sulfamethoxazole and amoxicillin, is common and has been described in Niger (Langendorf, 2015). The mechanisms of acquisition of this antibiotic resistance can differ: production of enzymes which destroy or modify the antibiotic agent, which often occurs secondary to exchange of genetic material, such as plasmid transfer; modification of the antibiotic target; and reduction in bacterial membrane permeability to the antibiotic (Blair, 2015).

Resistance to ciprofloxacin is generally caused by the accumulation of mutations in the *gyrA* gene, which encodes for one of the DNA gyrase subunit, the main target of quinolone in gram-negative bacteria, as well as the *par* genes encoding topoisomerase IV (Jacoby, 2005; Redgrave, 2014). Such accumulations would be expected after prolonged courses of antibiotic treatment. Plasmid-mediated acquisition of quinolone resistance has also been described. Although the quinolone resistance genes contained on these plasmids generally mediate only a small increase in the minimal inhibitory concentration of quinolones, these changes are sufficient to facilitate the selection of mutants with higher levels of resistance (Strahilevitz, 2009).

The ciprofloxacin that will be used in this study will be supplied by MSF-Logistique, Mérignac, France, using suppliers that have been prequalified by the WHO. The oral suspension is produced by Bayer Pharmaceuticals.

**Randomization procedures**

Overall, villages will be randomized in a 1:1:1 ratio to receive standard care, household-level prophylaxis or community-level prophylaxis.

When a HA has met inclusion criteria in the study, a set of sequentially-numbered sealed envelopes will be prepared, each containing the name of the intervention arm. When the first case presents from a village after the study has opened in the HA, study staff in the health center will telephone the person responsible for keeping the randomization lists, who will be otherwise independent of the study functioning.

The central randomizer will keep a master list of names of villages and their study assignments. A similar list of village names and study assignments will also be produced in each health center as villages are included. When subsequent cases present to the same health center, study staff based in the health center will verify whether the village in question has already been randomized. This information will in turn be double-checked against the central-level randomization list.

Because of the unpredictability of the villages that will notify meningitis cases and be included in the study, a randomization list will not be prepared in advance, so as to ensure a balanced randomization.

After a HA has been included in the study, the first case reported in a village will trigger the study intervention in that village. In villages randomized to community-level prophylaxis, distribution of ciprofloxacin will occur only once, after the first notified case. In villages which have been randomized to household-level prophylaxis, prophylaxis of a notified patient’s household will occur with each subsequent case notified from that village. More details are provided below in the section entitled “Study interventions”.

**Meningitis surveillance and incidence calculations**

Once a HA has been included in the study, a case-based surveillance system will be put in place in health centers, or reinforced if one is currently in place in the study area.

Suspected cases of meningitis that present to health posts will be referred to the nearest health center. In each health center, the diagnosis and treatment of meningitis will follow national protocols.

In line with current international recommendations, the following case definitions will be used:

- Suspect case: Abrupt fever (>38.5°C rectal or >38.0°C axillary) and at least one of the following signs: neck stiffness, floppy neck, bulging fontanelles, convulsions or other meningeal signs.
- Confirmed case: Isolation or identification of a causative organism (*Neisseria meningitidis, Streptococcus pneumoniae, Haemophilus influenzae serotype b*) in the CSF of a suspect case by culture, PCR or agglutination test.

For each suspected case presenting to a health center or district hospital, the following information will be recorded: Age, sex, village of origin, vaccination history, treatment(s) received, presence of sequelae, results of field-based laboratory tests, outcome. A unique identifier will be assigned following the national recommendations for case-based meningitis surveillance. In the setting of the current trial, information about whether the suspected case had received a dose of ciprofloxacin will also be recorded on the standardized forms.

Lumbar puncture and CSF analysis will occur following standard protocols and procedures. Any information collected as a result of this procedure and potential tests will be recorded in the national surveillance system in place. All clinical care will be identical between study arms.

Once the first case has been notified from a village after the study has opened in a health area, a team of community health workers will perform a village census, so as to provide the most accurate possible information for the calculation of meningitis incidence. This census will happen in parallel, or even after, the study interventions described below.

**Target population**

All residents of included villages will be eligible for participation in the study.

For participants in the arms which receive ciprofloxacin, the only persons ineligible to receive ciprofloxacin will be persons currently exhibiting symptoms consistent with meningitis (who will be immediately referred to the nearest health center) and persons with a known allergy to fluoroquinolone antibiotics.

**Study sites**

Study activities will take place in the District Hospital of the Health District in question, as well as in the health centers of HA included in the study. Each health center will have its usual complement of clinical and paraclinical staff necessary during an outbreak. Each health center will also have dedicated study staff whose roles are defined below.

Additional study activities will take place in the villages and in participants’ homes. All distributions of ciprofloxacin, whether household-level or community-wide, will happen in the community. Participants in the resistance sub-study will collect their samples at home and return them to study personnel in their communities. (See below for further detail.)

**Study procedures**

*Interventions*

In the standard care arm, after the first case has been notified from a village, a study nurse will visit the village with a local community member. The study nurse will lead an informational session for members of the community regarding the signs and symptoms of meningitis, and the urgency of presenting to the nearest health center as soon as possible if any of those signs or symptoms arrive.

In the household-level prophylaxis arm, each time a case is reported from a village that has been randomized to this arm, a study nurse will visit the household of the notified case within 24 hours. The study nurse will offer ciprofloxacin to all persons currently living in the same household compound and present at the time of the visit. Household members who have accompanied the notified case will be offered ciprofloxacin at the health center / district hospital. All doses of ciprofloxacin will be directly observed. If a household member is absent during the study nurse visit to the household, a return visit will be arranged.

In this study, a household will be defined as a group of people living in the same building or group of adjacent buildings and under the authority of a single head of household. In the case of polygamy, all wives (and their children) of a male head of household will be considered to members of a single household, provided that they live in the same building or group of adjacent buildings. In the case of multiple heads of household living in one compound of houses, only persons living under the authority of the head of household of the declared meningitis case will be considered a household member and therefore invited to take ciprofloxacin.

In the community-level prophylaxis arm, after the first case is reported from a village that has been randomized to this arm, a member of study staff will visit the village within 24 hours. During this visit, arrangements will be made for a community-wide distribution of ciprofloxacin, preferably within 72 hours of the initial case presentation at the health center / district hospital. These distributions will be organized in collaboration with local administrative and health authorities, such as village chiefs and community health workers, and will take place after a series of community meetings explaining the study and the nature of the distribution to the inhabitants of the village. A sensitization plan will be agreed in each village. All doses of ciprofloxacin will be directly observed.

*Dosing and administration of ciprofloxacin*

Oral ciprofloxacin will be dosed based on age following the guidelines set forth in the table below. Tablets will be administered with water. Children unable to swallow pills will have the tablet crushed and dissolved in sweetened water. The oral suspension will be prepared according to manufacturer’s instructions.

**Table 1: Age-based dosing of ciprofloxacin**

| Age | Dose (mg) | Formulation |
| --- | --- | --- |
| >12 years | 500 | 1 tablet |
| 5-12 years | 250 | 1 tablet |
| 1-4 years | 125 | ½ tablet (250 mg tablet) |
| 3-11 months | 100 | 2 ml oral suspension (250 mg/5ml) |
| <3 months | 75 | 1.5 ml oral suspension (250 mg/5ml) |

**Standard care for meningitis**

Throughout the study period, the diagnosis and treatment of meningitis in all health centers in study areas will be provided free of cost to any person, regardless of whether they have participated in a distribution of ciprofloxacin. Health center staff will provide physical examinations, lumbar punctures if necessary, and appropriate antibiotic therapy following national protocols. All patients in study areas who need referral from a health center to the district hospital will be transported free of charge.

Participation in the study will in no way affect the eligibility of a village or a HA for reactive vaccination with a polysaccharide or conjugate vaccine.

**Sample size**

The sample size needed to show a difference in the reduction of the meningitis attack rate will be predicated on multiple factors, some of which can be reliably estimated (risk of developing meningitis among household contacts of cases), and others which cannot be reliably estimated, or which will depend on the study area. The overall size of the epidemic (as measured by cumulative incidence) is not reliably predictable. Average household size and average village size also vary significantly by region of Niger.

Table 2 presents different simulations of the number of clusters necessary given specified attack rates and impacts of the ciprofloxacin intervention under the following hypotheses, using the method detailed by Donner and Klar (1996):

- Risk of type 1 error (alpha) of 5%
- Power of 90%
- Inter-cluster correlation coefficient of 0.025
- Average village population of 300 persons

**Table 2 : Number of clusters necessary given different attack rates and intervention effectiveness**

| *Reduction in attack rate due to intervention* |  | *Meningitis attack rate in arm receiving standard of care (%)* | | | | | |  |
| --- | --- | --- | --- | --- | --- | --- | --- | --- |
|  |  | 0.5 | 1 | 2 | 3 | 4 | 5 | |
| 50% |  | 355 | 177 | 88 | 58 | 44 | 35 | |
| 70% |  | 157 | 79 | 39 | 26 | 20 | 16 | |
| 90% |  | 81 | 41 | 20 | 14 | 10 | 8 | |

For example, in the situation where the end-of-epidemic attack rate in villages with at least one case is 1%, and the reduction of the attack rate due to ciprofloxacin is 70%, a total of 79 villages would need to be randomized in each arm of the study.

Because of these uncertainties, the overall target sample size necessary will be set after four weeks of accumulated study data. This period is sufficiently long to allow for a description of the demographic co-factors in the study area. Table 2 shows the high variability given these co-factors, but the most likely combinations of scenarios present sample sizes that are logistically feasible.

**Resistance sub-study**

A total of 200 participants in the standard care arm and 200 participants in the community-level prophylaxis arm will be offered enrollment in the resistance sub-study. In this sub-study, a total of 3 stool samples will be collected from each participant, at Day 0, Day 7 and Day 28. Samples from Day 0 in the village-wide distribution arm will be collected prior to distribution of ciprofloxacin.

Written consent will be obtained from participants, or their parents/guardians, for participation in the resistance sub-study.

To facilitate the collection and prompt processing of the stool samples, they will be collected from 20 participants in each of 20 villages (10 villages in the standard care arm, 10 villages in the community-level prophylaxis arm.) In each of the arms, the resistance sub-study will begin once at least 10 villages have notified cases after the study start.

*Selection of villages*

Because of laboratory capacity, a maximum of two villages per day will be included in the resistance sub-study. Once the resistance sub-study has begun, the first odd-numbered village which notifies a case each day in each of the two arms will be included in the sub-study.

*Selection of participants*

In each village selected to participate in the sub-study, 20 households will be randomly selected from an exhaustive list of households in the village prepared specifically for this purpose. One member of each household will be randomly selected and will be invited to participate in the resistance sub-study. The only exclusion criteria will be if the participant knows in advance that they will be absent during the follow up visits at days 7 and 28. If this is the case, a second member of the household will be randomly selected as a replacement. Written informed consent will be obtained from each participant or their parent/guardian for participation in the resistance sub-study. Each participant will be given a unique identifier and asked whether or not they received the ciprofloxacin distribution.

*Sample collection*

In villages selected for the resistance sub-study (in both the standard care arm and community-level prophylaxis arm), participants will be identified at the time of the community sensitization messages being delivered by a study nurse in conjunction with a laboratory technician. A stool sample container will be distributed to each participant, with instructions on how to collect the sample the following morning. Study staff will then return to the village the following morning to collect the fresh stool samples. The same procedures will be followed on days 7 and 28, with one visit to provide the sample container and a second visit to pick up the fresh samples the following morning. Day 0 samples will be collected up until the time of ciprofloxacin administration in the village. At day 7 and 28, samples will also be accepted the day following the scheduled sample collection (i.e., days 8 and 29), if the participant is not able to provide a stool sample on the scheduled day.

*Sample processing*

The processing of stool samples will depend on the location of the study and its distance from the principal study lab in Maradi. The processing of samples will be detailed in the study SOPs. In brief, stool samples will be collected by study staff and either stored at 4°C or inoculated in a transport medium until reception in the central lab. Stool samples will then be plated on a MacConkey agar plate containing ciprofloxacin. After incubation, colonies will be identified using standard microbiological methods. Antibiotic resistance profiles will be determined using the disk diffusion method. The minimum inhibitory concentration (MIC) of relevant antibiotics will be determined. All identified strains will be stored in the Epicentre lab at Maradi. A total of 10% of samples will be sent to a reference laboratory for quality control purposes.

*Participant retention*

Once a participant has been included in the resistance sub-study, the study team will make every reasonable effort to ensure his or her participation for the entire study period. It is projected that attrition rate will be 25% and that up to 20% of samples will be inappropriately conserved and therefore be not able to be analyzed. The sponsor will be responsible for developing study SOPs to achieve this level of follow-up. Soap will be provided with each stool sample container so as to give sensitization messages about handwashing and to ensure that participants are able to wash their hands after sample collection (see Reimbursement below).

*Sample size considerations*

Assuming a baseline prevalence of enterobacteriaceae resistant to ciprofloxacin in the community of 20%, over three repeated visits, a sample size of 131 persons in each of the two arms would have 90% power to detect a change in the prevalence to 30%. Assuming an attrition rate of 25% and 20% of improperly collected samples, a sample size of 200 persons per arm would allow for the calculation of the primary resistance objective with sufficient power.

**Data analysis**

The primary analysis of the difference in attack rate by study arm will be based on the overall meningitis incidence during the epidemic in villages of each arm after inclusion in the study, taking into account the cluster-randomized nature of the trial. Pairwise comparisons will be made between each intervention group using the student’s t-test. ANOVA and multiple linear regression will be used to compare the attack rates between the three study groups, and will be expressed in hazard ratios with 95% confidence intervals. The sponsor will be responsible for developing a written analytic plan.

The analysis of the resistance sub-study will be done on the basis of the per-protocol population for whom all three stool samples are available. Acquisition rates at 7 and 28 days following the initial sample will be calculated with corresponding 95% confidence intervals using the binomial distribution. The random selection of participants and households nested into a cluster-randomized design will be taken into account.

All P values will be 2-sided with *p* < 0.05 considered statistically significant. All other analyses of secondary analyses would be best regarded as exploratory, and any significant findings for these endpoints would need to be confirmed with further studies. Missing data will be assumed to be missing at random.

**Data collection, management and quality assurance**

All the information required by the study protocol will be entered on standardized CRFs and surveillance worksheets in French. The Field Investigator will validate all CRFs and surveillance worksheets for completeness and accuracy, signing and dating each to attest to his/her responsibility for the quality of all data recorded and that the data represents a complete and accurate record of each child’s participation in the study. During the study, all CRFs and surveillance worksheets will be maintained at the central study office in a secure location to ensure confidentiality. All validated CRFs and surveillance worksheets will be double-entered, compared and verified for accuracy.

A data validation plan will be prepared before study initiation. Errors will be detected by programs designed to detect missing data or specific errors in the data. These errors will be summarized along with detailed descriptions of the specific problem in a Data Query Report, which will be sent to the Field Investigator for resolution. Written documentation of changes will be available via electronic logs. The primary sponsor will be responsible for maintaining study databases.

**Timeline**

The estimated total duration of the study is one meningitis epidemic season. Given Epicentre and MSF’s established field presence in Niger and experience with performing research in emergency settings, we anticipate 1 month will be used for study preparation, including development of SOPs and ethical/administrative clearance. Once an epidemic has been declared in an eligible district, we anticipate 1 week will be used for recruitment and training of field personnel, and putting in place a facility-based surveillance system. The length of recruitment time will depend on the dynamics of the epidemic, but under the most reasonable assumptions described in Table 2, it should be feasible to include the necessary number of villages over the course of an average-sized epidemic. Analysis and write-up will be completed in the 3 months after study closure.

**SAFETY**

**Documenting serious adverse events**

Passive surveillance for serious adverse events will be conducted from study beginning through 28 days following the last dose of ciprofloxacin administered (whether in the household-level or community-level prophylaxis arm). This surveillance will cover all study participants and be carried out at the health facility-level by study staff.

Serious adverse events (SAEs) are defined as any untoward medical occurrence that at any dose of medicine received results in death, is life threatening, requires inpatient hospitalization or prolongation of existing hospitalization, or results in persistent or significant disability or incapacity, or any medically important event / reaction that may jeopardize the subject and may require medical or surgical intervention to prevent one of the outcomes listed above (Bonhoeffer 2002 and 2004).

SAEs will be defined as an adverse event that at any dose of ciprofloxacin that

1. results in death;
2. is life threatening;
3. requires inpatient hospitalization or prolongation of existing hospitalization;
4. results in persistent or significant disability or incapacity
5. results in congenital malformation or birth defect
6. is medically important event / reaction that may jeopardize the subject and may require medical or surgical intervention to prevent one of the outcomes listed above

Persons who die in a health facility will have events before and at the time of death transcribed from hospital records to determine the cause of death. If death occurs outside of the hospital, cause of death will be established using a standard verbal autopsy form administered to determine the clinical picture and treatment received before death.

SAEs identified will be reported to the Field Investigator within 24 hours of becoming aware of the SAE, whether or not it is considered to be associated with a study intervention. The field team will assemble specific documentation, including medical records and other supporting documents, and will record on the CRF: type of SAE; description of event with time of onset in relation to vaccination and severity; and any medical actions taken and outcome. Where applicable, hospital records and verbal autopsies should be obtained. The dossier will be submitted to the Principal Investigator and Study Monitor for adjudication and determination whether the cases were ciprofloxacin-related. In case of discrepancy between the Principal Investigator and the Study Monitor regarding the causal relationship of ciprofloxacin to an SAE, the Sponsor will designate an independent third party to adjudicate the relationship. All SAEs will be followed up until the event resolves, stabilizes, or is otherwise explained. If an SAE remains unresolved at the time of study closure, a clinical assessment will be made by the Field Investigator and sponsor to determine if continued follow up of the SAE is warranted. All relevant field personnel will be appropriately trained in the reporting and treatment of serious adverse events according to GCP. The National Ethical Committee of Niger will receive a quarterly status report including all notified SAEs during the study. Copies of each report and documentation of IRB notification will be kept in the Trial Master File by the Field Investigator.

**Assessment of causality**

All SAEs within the first 28 days post-administration of ciprofloxacin will be assessed for a causal relationship with ciprofloxacin by the Principal Investigator / designee. Every effort will be made by the study team to explain each event and assess its causal relationship, if any, to administration of ciprofloxacin. Appropriate medical judgment will be used to determine the causal relationship, considering all relevant factors including the pattern of reaction, temporal relationship, re-challenge, biological plausibility, and confounding factors such as concomitant medication, concomitant disease and relevant history.

The likelihood of the relationship of the event to ciprofloxacin will be recorded as follows:

**Related,** when there is a reasonable possibility that ciprofloxacin contributed to the event.

**Unrelated,** when administration of ciprofloxacin is not suspected to have contributed to the event (CIOMS, 2003).

All post-administration serious adverse events (e.g. those occurring within 30 minutes of administration) will be considered related to ciprofloxacin.

**MONITORING**

The sponsor will permit trial-related monitoring and Institutional Review Board review, providing direct access to source data and documents.

A qualified and appropriately-trained Study Monitor will be designated by the sponsor to carefully monitor all aspects of the study.

The Study Monitor will perform an initial audit before the start of the study to ensure all necessary tools and supports are in place for study implementation. During the project period, the Study Monitor will routinely contact study sites and perform on-site visits to inspect facilities and documentation, observe performance of study procedures, discuss the protocol in detail and identify and clarify any areas of weakness. The extent, nature and frequency of site visits during the project period will be based on considerations of study objectives, study design and complexity, and enrollment rate.

Monitoring will be conducted according to good practice guidelines and study SOPs. The Study Monitor will have access to all records necessary to ensure the integrity/validity of the recorded data and will periodically review the progress of the study. During site visits and contacts, the Study Monitor will specifically:

1. Check and assess the progress of the study

2. Review study data collected

3. Perform source data verification to verify compliance with study SOPs

4. Verify compliance with human subjects protection and research guidelines

5. Identify any issues and discuss possibilities to address their resolution with the Sponsor and Field Investigator

This will be done in order to verify that:

1. The data are authentic, accurate and complete

2. The safety and rights of participants are being protected

3. The study is conducted in accordance with the approved protocol (and any subsequent amendment), and all applicable administrative requirements

**ETHICAL CONSIDERATIONS**

**Ethical considerations particular to cluster-randomized trials**

A variety of ethical questions should be considered when designing and implementing cluster-randomized trials. These questions include who is considered a research subject; from whom, how and when informed consent should be obtained; whether clinical equipoise exists; the determination of risks and benefits; the protection of vulnerable groups; and the roles and responsibilities of “gatekeepers” (Weijer, 2011).

A series of recommendations for the ethical design and conduct of cluster randomized trials was developed by a consensus group in 2012, referred to as the Ottawa Statement (Weijer, 2012). Several flaws have been identified in the original statement, particularly regarding the definition of study subjects and the disclosure of randomization (van der Graaf, 2015).

The literature surrounding the ethical considerations of cluster-randomized trials is biased towards developed-country settings. The conduct of such trials in sub-Saharan Africa is less well-represented in the literature. This is important both because the setting and contexts are different, but also because the types of intervention are often quite different from those seen in the developed world. One recent description from a cluster-randomized trial carried out in schools in Kenya describes a number of logistical and ethical difficulties, particularly regarding community involvement and informed consent (Okello, 2013).

The setting of the proposed cluster-randomized trial poses some challenges. Conducting research during an emergency such as a meningitis outbreak is challenging, particularly given the fear that a meningitis outbreak can provoke in the population.

Two general types of cluster-randomized trials have been described (Sim, 2012). The first is a “cluster-cluster” trial, where the intervention necessarily takes place at the cluster level (such as treatment of a community well). The second is an “individual-cluster”, where the intervention takes places at the individual or household level. The proposed trial is an “individual-cluster” trial in the paradigm described by Sim, which necessitates the special consideration of the role of political and administrative authorities as gatekeepers, as well as the nature of individual consent for participation.

The following sections will address some of the important ethical considerations of the proposed trial design, taking into account the guidance of the Ottawa Statement and other important documents from the medical/ethical literature on the subject of cluster-randomized trials.

**Summary of known and potential risks, identification of vulnerable populations**

There is no data on the use of ciprofloxacin as an epidemic response in the meningitis belt of sub-Saharan Africa. However, based on documentation of safety of its use in multiple other settings, the individual risks associated with its use as a single-dose prophylactic treatment in this trial are expected to be minimal, including among pregnant women and children, as described above.

One potential community-level risk associated with the use of ciprofloxacin as prophylaxis is the development of enterobacteriaceae resistant to fluoroquinolones. In the event of a positive primary outcome, no recommendation on the routine use of ciprofloxacin in similar settings would be given without a description of the potential effects of the strategy on antibiotic resistance. The resistance sub-study seeks to respond to this open question and quantify the risk, if any, associated with single-dose ciprofloxacin.

All clinical procedures (administration of ciprofloxacin and preparation of stool samples) will be performed by adequately trained and experienced personnel under regular supervision to minimize any risk or discomfort to participants. Resistance sub-study participants may have a higher risk of fecal-oral contamination. To combat this risk, sub-study participants will be provided soap before each sample collection and instructions on handwashing after defecation and sample collection.

Given the difficult-to-predict evolution of meningitis epidemics, it is also possible that the study would not reach its target sample size, and therefore not have sufficient power to evaluate the primary outcome. This could happen because the epidemic naturally does not reach a large enough magnitude, or because a reactive vaccination campaign is undertaken in some or part of the study area. No reactive vaccination will be stopped or delayed due to the implementation of this study.

**Risk minimization and benefits**

All personnel involved in collecting and processing biological samples are trained health care personnel, who will be provided with additional training to avoid or minimize the possibility of any unplanned side effects of these procedures.

The direct benefits individual participants may expect from participating in this study include receiving information about the signs and symptoms of meningitis and information about the free nature of health care during such an epidemic. All participants may also expect to benefit from contacts with members of the study team and the assurance of the best available medical care with close and regular surveillance. Recipients of ciprofloxacin may also potentially benefit from the possible protective effect of the antibiotic against the subsequent development of meningitis. At the population level, an important benefit of obtaining data on the efficacy of the use of ciprofloxacin in response to an epidemic, if proven protective, is the antibiotic potentially being made available for a larger population, particularly in light of current shortages of vaccine against NmC.

To minimize the risk of not reaching the target sample size, starting criteria have been set forth to reduce the possibility of starting the trial too early in an epidemic. Given that reactive vaccination remains the recommended epidemic response for meningitis, all study areas would be evaluated for reactive vaccination by the local health authorities following the usual procedures in place in Niger, and would be eligible for reactive vaccination campaigns. Nonetheless, reactive vaccination campaigns often take 6-8 weeks to organize, so if the proposed study is put into place early in the epidemic, the amount of study inclusion time without this potential confounding factor will be maximized.

**Definition of research subject and identification of gatekeepers**

Given that the primary outcome of interest is the overall meningitis incidence in the three different arms, all residents of randomized villages should be considered research subjects (Weijer, 2012; van der Graaf, 2015). Although there are certain unknowns regarding the sample size necessary for the evaluation of the primary outcome (see section “Sample Size” above), even when using conservative estimates, we could reasonably expect at least 20 000 persons to be living in the study area of each arm, therefore a total of at least 60 000 research subjects under the current definition.

The logical “gatekeepers” as described by the Ottawa Statement for the proposed trial are the health and political/administrative leaders of the study areas in question. For the purposes of this study, the gatekeepers who will be asked for permission will be the doctor or nurse in charge of the Health Center in a given HA, as well as the village chief and his/her deputies for the villages eligible for inclusion.

**Informed consent**

The role of gatekeepers in the proposed trial is extremely important, but their permission for participation in the trial will not be considered proxy consent for an individual’s participation in the trial (Weijer, 2012). Nonetheless, following the Ottawa Statement, given that the proposed trial would substantially affect the interests of the cluster (village, HA), we will obtain the permission of the identified gatekeepers in each HA and village eligible for inclusion in the trial. This permission will be documented following the traditional format of an informed consent procedure for inclusion in a study. The process of obtaining permission for randomization will consist of the explanation and discussion of an informational notice (Appendix A) about the purpose of the study, the study interventions, procedures to be followed and the risks and benefits of participation, both for the HA/village as a whole and for individual participants. The informational notice, the discussion and written permission document will be reproduced in the local language. At the village level, at the end of this process, the village chief will be asked to sign the permission document for the village.

In the arms which will receive ciprofloxacin distribution (either village-wide or household-level) it will not be possible to obtain written informed consent from each individual who receives ciprofloxacin. As described above, it is likely that over 20 000 people will be included in the village-wide prophylaxis arm, and in the household-level prophylaxis arm, several thousands are also likely to be eligible to receive ciprofloxacin. In the setting of a public health emergency, obtaining written informed consent from each participant is simply logistically impossible.

As detailed by Sim, written informed consent of each participant in a cluster-randomized trial should not be considered an absolute requirement (Sim, 2012), and the context of the proposed trial meets the conditions set out for this. The waiver of individual-level consent is also foreseen in the Ottawa Statement (Weijer, 2012). Nonetheless, as pointed out by Sim, “Although informed consent may not be sought in a cluster-randomized trial, the goals that it seeks to achieve may nonetheless by protected by other means.”

Therefore, in villages randomized to household-level prophylaxis, when the study nurse visits an eligible household, he or she will present the study, its interventions, procedures to be followed and the risks and benefits of participation following the text presented in the informational notice for participants (Appendix B). The members of the household will be given opportunity to ask questions and only those willing to take ciprofloxacin will be administered the drug.

In villages randomized to village-wide prophylaxis, a series of community informational sessions will be organized in advance of the distribution. A text (Appendix B) that presents the study, its interventions, the procedures to be followed, the voluntary nature of participation and the risks and benefits of participation will be read during this meeting, and community members will be given the opportunity to ask questions. The logistical setup of these informational sessions will vary depending on village size and other factors, but the goal will be to have one session for each 25 households, as to allow for better participation. Similarly, during the sensitization campaigns prior to the distribution, the voluntary nature of participation will be underlined. Those people who present to the distribution site will therefore be considered to have been duly informed of the risks and benefits of their taking ciprofloxacin and the voluntary nature of their participation.

The plan outlined in the above two paragraphs is in line with the Ottawa Statement and the criteria set out by Sim.

Written informed consent will be sought prior to inclusion in the resistance substudy. Prior to collection of the first sample, the study nurse will read aloud in the local language the informational notice and informed consent form for the substudy (Appendix C). This informational notice will include information on the purpose of the sub-study, procedures to be followed and the risks and benefits of participation. Study staff will give the participant or their parent/guardian ample opportunity to inquire about details of the study and ask any questions. The notice will be read to the participant in the presence of a literate and impartial witness. The witness will sign and date the consent form to attest the consent process appears to be fair and the participant (or their parent/guardian) voluntarily accepts to participate.

One copy of the Informed Consent Form will be kept on file by the Field Investigator. The participant will receive the Information Sheet and a second copy of the signed and dated Informed Consent Form.

**Confidentiality**

The primary objective of the study concerns population-level meningitis incidence. The case-based surveillance that will be put in place in the health centers will require that the names of meningitis cases be recorded case reporting forms (following national procedures).

When the individual-level information for each case is entered into the database, only the unique identifier generated by the national system will be entered. No nominative information will be saved in an electronic format.

Participants in the resistance substudy will be assigned a unique individual identification number that contains no personal identifiable information. This unique ID will be used in all CRFs and laboratory specimens for the substudy. All records that contain names or other personal identifiers, such as locator forms and informed consent forms, will be stored separately from study records identified by code number. Substudy participants and/or their caregiver will be informed that representatives of the sponsor or ethical committees may inspect their medical records to verify the information collected, and that all personal information made available for inspection will be handled in the strictest confidence. Participants’ individual information will not otherwise be released outside of the study. Personal identifiers will not be included in any study report.

All study records and data will be kept confidentially under lock and key and/or electronic password protection, as appropriate and in accordance with local data protection laws for 5 years. Only senior study personnel will have access to these records.

**Reimbursement**

Participants will not be paid for their participation in the study. Participants in the resistance sub-study will be provided with soap prior to each stool sample collection to ensure their ability to wash their hands after collecting their sample.

**Storage of specimens**

Stool samples collected for the resistance substudy will be destroyed within three months of processing.

**Institutional Review Board approval**

The study will be submitted to the research ethics committee of the Ministry of Health Niger, and the Ethical Review Board of Médecins Sans Frontières for their approval, and will be done in accordance with the Declaration of Helsinki and ICH guidelines.

**Declaration of conflict of interests**

The primary and secondary sponsors declare no conflict of interests.

**STUDY ADMINISTRATION**

**Protocol amendments**

Any modifications to the protocol which may impact the conduct of the study or may affect patient safety / benefit, including changes of study objectives, study design, patient population, sample sizes, and study procedures, will require a formal amendment to the protocol. Such amendment will be agreed upon by the sponsors and approved by the appropriate ethics committee prior to implementation. The sponsor will be responsible to notify the trial registry. Administrative changes of the protocol, including minor corrections and/or clarifications that have no effect on the way the study is to be conducted, will be agreed upon by the sponsors and will be documented in a memorandum. The relevant ethics committees will be informed of the study location when it has been identified and of the target sample size when it is set.

**Protocol deviations and violations**

A protocol violation is any departure from the approved protocol, trial documents or any other information relating to the conduct of the study which may affect the safety of trial participants or the study outcomes. Examples include failure to obtain informed consent (i.e. no documentary evidence) for participants in the resistance substudy or administration of ciprofloxacin to participants that do not meet inclusion/exclusion criteria.

A protocol deviation is any departure from the approved protocol, trial documents or any other information relating to the conduct of the trial that does not result in harm to the trial participants and does not significantly affect the study outcomes. Examples of deviations include a follow-up visit for the resistance substudy outside the study visit window or an isolated incident of a missed or incomplete study procedure. Serious or repeated protocol violations or deviations will require assessment of the root cause and implementation of corrective and preventive action plans. They may constitute grounds to interrupt the trial.

Any changes from protocol-specified procedures and study-related SOPs occurring during the conduct of the trial will be documented and reported as protocol violations or deviations. Protocol deviations and violations will be monitored by the Field Investigator/Study Monitor and immediately reported to the sponsor. The sponsor will inform reviewing ethical committees, as appropriate and in accordance with the requirements of the involved committees.

**Ancillary care and insurance**

In the event that a participant suffers injury attributable to participation in this study, appropriate medical management and treatment will be paid for by the study and provided by the local health authority with support from the study staff. The study sponsor will have insurance to cover non-negligent harm associated with the protocol.

**Data storage and archival**

The sponsor will provide the Field Investigator and Study Monitor with a Trial Master File, which will be used to file the protocol, correspondence with the IRB and sponsor, and other study-related documents. The Field Investigator and Study Monitor will maintain, and store securely, complete, accurate and current study records throughout the study.

The sponsor will keep essential documents, including participant’s medical records and CRFs, until at least 5 years after study closure. No data will be destroyed without the permission of the sponsor.

**Dissemination and authorship policy**

When the trial report is completed, the investigators will share the summary results with local, regional and national health authorities and answer any questions.

The findings from this study will also be published in a peer-reviewed scientific journal and disseminated at appropriate national and international conferences. The ultimate decision to submit a manuscript will remain with the primary sponsor. Every attempt will be made to reduce to an absolute minimum the interval between the completion of data collection and the release of the study results. Trial results will be disseminated to key stakeholders regardless of the direction or magnitude of effect.

The sponsors will determine the specific topics and numbers of publications, with rights to authorship being determined by intellectual contribution to the study design, implementation, and analysis, as is specified by most major scientific journals. Preference will be given for publication in peer-reviewed, open-access journals with appropriate readership and high impact factors.

**Data sharing policy**

The research data will be the property of the sponsors, though we realize that the data collected from this study may provide other investigators with the opportunity to answer other scientific questions. Therefore, data will be made as widely and freely available as possible, in a timely fashion, while safeguarding the privacy of participants and protecting confidential data. A de-identified data set can be made available under a data sharing agreement that provides for a commitment to using the data only for research purposes and securing data using appropriate technology.

**STUDY MANAGEMENT**

**Study sponsors**

The primary sponsor will develop the study protocol, with substantial input from MSF and other partners. The primary sponsor will hold the data and conduct all analyses. The final report will be written by the primary sponsor, who will have full access to the data and final responsibility for the data analysis and decision to submit for publication. Primary sponsor staff will independently monitor study execution at field sites.

The secondary sponsor is Médecins Sans Frontières- Operational Center Geneva (MSF-OCG). MSF-OCG has agreed with the primary sponsor to act as the Primary Sponsor’s legal representative in relation to the trial site and provide funding for the trial.

**Scientific Committee**

A Scientific Committee (see Appendix D for Terms of Reference and Membership) will be asked to contribute to the following activities:

- Review drafts of the trial protocol, and agree on a final version;
- Advise and agree on an Analysis Plan covering data generated by the trial;
- Reply promptly to specific queries regarding the trial methods, practical implementation, analysis and interpretation;
- Review drafts of the final trial report, and agree on a final version.

Reporting procedures of the Scientific Committee to the sponsor are as outlined in the Terms of Reference and Membership (Appendix D). The Sponsor Study Director assumes responsibility that the Scientific Committee is informed of all components of the trial protocol.

**Human resources**

Overall study development and direction will be provided by a team of 4 investigators based in Paris, Geneva and Niger (Figure 1). Rebecca Freeman Grais will serve as the Sponsor Study Director for the study. Dr. Grais is an international expert in vaccination in sub-Saharan Africa, and as the Sponsor Study Director, will be the guarantor of the trial. Matthew Coldiron, Sponsor Principal Investigator, is a medical epidemiologist with extensive experience in conducting research in resource-poor settings in sub-Saharan Africa. Dr. Coldiron will be responsible for ensuring appropriate study design and implementation of study procedures. Gabriel Alcoba, Co-Investigator, is the Tropical Medicine Advisor for MSF-OCG, and a paediatrician specialized in p. Dr. Alcoba will be responsible for ensuring that the study protocol is well-integrated into the medico-operational response to the outbreak. The Field Investigator will be a medical doctor with experience in the conduct of clinical trials in sub-Saharan Africa.

Day-to-day study activities will be carried out in the villages reporting meningitis cases, in the health centers of the HAs reporting cases, in the District Hospital, and at the Epicentre laboratory in Maradi. These activities will be primarily carried out by the study field team.

The field team will be comprised of a Field Investigator and specialized health care personnel based in sites throughout the study area, who will be responsible for carrying out the study interventions, meningitis surveillance, and the collection of resistance sub-study samples. The Field Investigator will be under the supervision of the Sponsor Principal Investigator. There will be at least daily communication by email, teleconference or videoconference to discuss study activities and challenges with the Field Investigator and Sponsor Principal Investigator.

The Field Investigator will be responsible for coordination of all field worker and supervisor schedules, standardization of study interventions and assurance that activities are conducted according to protocol. The Field Investigator will supervise two teams with the following specific roles:

- Facility-based medical team: This group will include the normal staff of the health center or hospital, as well as dedicated study staff. At least one study nurse will be assigned to each health center, and will be responsible for carrying out the study interventions as necessary, including distribution of ciprofloxacin as described. For villages receiving community-wide prophylaxis, additional paramedical staff will be trained to carry out the distributions. At least one study data collection agent will also be assigned to each health center. This agent will be responsible for completing the data registers in each facility during the epidemic.
- Resistance sub-study teams: This group will include at least two dedicated study nurses and two dedicated laboratory technicians. They will be responsible for carrying out the resistance sub-study as described in the protocol, including obtaining informed consent from participants, distributing sample collection jars and sample return and stool sample processing in the field.

The supervisor of the Epicentre laboratory in Maradi will be responsible for overseeing stool culture, species identification and resistance testing, which will be carried out by at least two laboratory technicians based in the central lab.

Data will be entered at a central level by a data entry operator under the supervision of the field investigator.

Field teams will in all cases follow the security rules and regulations set forth by the MSF section supporting the MOH in its outbreak response. These rules and regulations will detail where, how and when staff may move about the study area.

**Training**

Study staff will be trained in GCP, serious adverse event guidelines, clinical assessment of patients, completion of relevant source documents and CRFs, specimen collection and storage of samples. Initial training will be provided by the Principal Investigator and Field Investigator with support from other partners. Refresher training will be provided on an ad hoc basis to all study personnel. Routine audits performed by the Study Monitor will be used identify procedures that need to be strengthened and reinforced in routine refresher training. Figure 1. Study organogram

*Scientific Committee*

Sponsor Study Director
Rebecca Grais

Primary Co-Investigator

Gabriel Alcoba

Epicentre Maradi
Laboratory technicians

Resistance sub-study teams

Study nurse

Study lab technicians

Facility-based medical teams

Study nurse(s)

Data collection agent

Central data entry operator

Epicentre Maradi Laboratory Supervisor

Co-Investigators

Céline Langendorf

Anne-Laure Page

Ali Djibo

Eric Adehossi

Dorian Job

Study monitor

Sponsor Principal Investigator
Matthew Coldiron

Field Investigator

**REFERENCES**

Blair JMA, Webber MA, Baylay AJ, Ogbulu DO, et al (2015). Molecular mechanisms of antibiotic resistance. *Nature Reviews Microbiology* 13:42-51.

Boisier P, Nicolas P, Djibo Saacou, Taha M-K, et al (2007). Meningococcal meningitis: Unprecedented incidence of serogroup X-related cases in Niger in 2006. *Clin Infect Dis* 44: 657-63.

Bonhoeffer J, Heininger U, Kohl K, Chen RT et al (2004). Standardized case definitions of adverse events following immunization (AEFI). *Vaccine* 22:547-50.

Bonhoeffer J, Kohl K, Chen R, Duclos P et al (2002). The Brighton Collaboration: addressing the need for standardized case definitions of adverse events following immunization (AEFI). *Vaccine* 22:298-302.

Burkhart JE, Walterspiel JN and Schaad UB. Quinolone arthropathy in animals versus children (1997). *Clin Infect Dis* 25:1196-204.

Coldiron ME and Salou H (2015). Description of an epidemic of meningococcal meningitis, Doutchi and Gaya Districts, Niger. Epicentre, unpublished report.

Conseil des Organisations Internationales des Sciences Médicales (2003). Lignes directrices internationales d'éthique pour la recherche biomédicale impliquant des sujets humains [cited 2015 December 28]. Available from: http://www.cioms.ch/publications/guidelines/french_text.htm.

Daugla DM, Gami JP, Gamougam K, Naibei N, et al (2014). Effect of a serogroup A meningococcal conjugate vaccine (PsA-TT) on serogroup A meningococcal meningitis and carriage in Chad: a community study. *Lancet* 383: 40-7.

Dellicour S and Greenwood B (2007). Impact of meningococcal vaccination on pharyngeal carriage of meningococci. *Trop Med Intl Health* 12: 1409-21.

Donner A and Klar N (2000). *Design and Analysis of Cluster Randomization Trials in Health Research*. London: Arnold.

Donner A and Klar N (1996). Statistical considerations in the design and analysis of community intervention trials. *J Clin Epidemiol* 49: 435-9.

European Medicines Agency (2014). EMA/CHMP Guidance document on use of medicinal products for the treatment and prophylaxis of biological agents that might be used as weapons of bioterrorism. 18 November 2014 CPMP/4048/01, rev.6

Ferrari MJ, Fermon F, Nackers F, Llosa A et al (2014). Time is (still) of the essence: quantifying the impact of emergency meningitis vaccination response in Katsina State, Nigeria. *Int Health*. 6:282-290.

Greenwood B. Meningococcal meninigitis in Africa (1999). *Trans R Soc Trop Med Hyg* 43:341-53.

Greenwood BM, Hassan-King M, Whittle HC (1978). Prevention of secondary cases of meningococcal disease in household contacts by vaccination. *BMJ* 1:1317-19.

Jacoby GA. Mechanisms of resistance to quinolones (2005). *Clin Infect Dis* 41 Suppl 2:S120-6.

Koutangni T, Boubacar Maïnassara H, Mueller JE (2015). Incidence, carriage and case-carrier ratios for meningococcal meningitis in the African meninigitis belt: A systematic review and meta-analysis. *PLoS ONE* 10(2):e0116725.

Kristiansen PA, Diomandé F, Ky Ba A, Sanou I, et al (2013). Impact of the serogroup A meningococcal conjugate vaccine, MenAfriVac, on carriage and herd immunity. *Clin Infect Dis* 56:354-63.

Langendorf C, Le Hello S, Moumouni A, Gouali M, et al (2015). Enteric bacterial pathogens in children with diarrhea in Niger: diversity and antimicrobial resistance. *PLoS One* 10(3):e0120275.

Lapeyssonie L (1963). La méningite cérébrospinale en Afrique. *Bull World Health Organ* 28 (suppl): 3-114.

McIntyre PB, O’Brien KL, Greenwood B, van de Beek D (2012). Effect of vaccines on bacterial meningitis. *Lancet* 380: 1703-11.

Mohammed I, Nasidi A, Alkali AI, Garbati MA et al. (2000). A severe epidemic of meningococcal meningitis in Nigeria 1996. *Trans R Soc Trop Med Hyg* 94: 265-70.

Molesworth AM, Thomson MC, Connor SJ, Cresswell MP et al. (2002). Where is the meningitis belt? Defining an area at risk of epidemic meningitis in Africa. *Trans R Soc Trop Med Hyg* 96:242-9.

Mueller JE and Gessner BD (2010). A hypothetical explanatory model for meningococcal meningitis in the African meningitis belt. *Int J Infect Dis* 14:e553-e559.

Nathan N, Rose AMC, Legros D, Tiendredeogo SRM et al (2007). Meningitis serogroup W135 outbreak, Burkina Faso 2002. *Emerg Infect Dis* 13: 920-3.

Okello G, Jones C, Bonareri M, Ndegwa SN, et al (2013). Challenges for consent and community engagement in the conduct of cluster randomized trial among school children in low income settings: experiences from Kenya. *Trials* 14:142.

Redgrave MS, Sutton SB, Webber MA and Piddock LJV (2014). Fluoroquinolone resistance: mechanisms, impact on bacteria, and role in evolutionary success. *Trends in Microbiology* 22:438-445.

Rosenstein NE, Bradley BA, Stephens DS, Popovic T, Hughes JM (2001). Meningococcal Disease. *N Engl J Med* 344:1378–88.

Sim J and Dawson A (2012). Informed consent and cluster-randomized trials. *Am J Pub Health* 3:480-485.

Stephens DS, Greenwood B and Brandtzaeg Petter (2007). Epidemic meningitis, meningococcaemia, and Neisseria meningitidis. *Lancet* 369: 2196–210.

Strahilevitz J, Jacoby GA, Hooper DC, Robicsek A (2009). Plasmid-mediated quinolone resistance: a multifaceted threat. *Clin Microbiol RevI* 22:664-89.

Sultan B, Labadi K, Guégan J-F and Janicot S (2005). Climate drives the meningitis epidemics onset in West Africa. *PLoS Med* 2:e6.

Trotter CL and Greenwood B (2007). Meningococcal carriage in the African meningitis belt. *Lancet Inf Disease* 7:797-803.

Van der Graaf R, Koffijberg H, Grobbee DE, de Hoop E, et al (2015). The ethics of cluster-randomized trials requires further evaluation: a refinement of the Ottawa Statement. *J Clin Epidemiology* 68:1108-1114.

Weijer C, Grimshaw JM, Eccles MP, McRae AD et al (2012). The Ottawa Statement on the ethical design and conduct of cluster randomized trials. *PLoS Med* 9(11): e1001346. doi:10.1371/journal.pmed.1001346.

Weijer C, Grimshaw JM, Taljaard M, Binik A, et al (2011). Ethical issues posed by cluster randomized trials in health research. *Trials* 12:100. doi:10.1186/1745-6215-12-100.

Whittle H, Evans-Jones G, Onyewotu G, Adjukiewicz A et al (1975). Group-C meningococcal meningitis in the northern savanna of Africa. *Lancet* 305: 1377.

World Health Organizaton (2014). Revised guidlines for meningitis outbreak response in sub-Saharan Africa. *Weekly Epidemiol Review* 89: 577-88.

World Health Organization (2015). Serogroup C in the meningitis belt: Facing the challenge. Meeting report.

Zalmanovici T, Fraser A, Gafter-Gvili A, Paul M, Leibovici L (2013). Antibiotics for preventing meningococcal infections. *Cochrane Database Syst Rev* 10:CD004785. doi: 10.1002/14651858.CD004785.pub5.

**APPENDIX A: Village leader informational notice and written permission document**

**Informational notice for village chiefs: Cluster-randomized trial of ciprofloxacin for contacts of cases of meningococcal meningitis as an epidemic response**

Madam, Sir,

Médecins Sans Frontières (MSF) is an independent, medical humanitarian organization providing health care in Niger. Epicentre carries out research studies supporting the Ministry of Health and MSF in Niger. We are currently supporting the Ministry of Public Health in its response to an ongoing meningitis epidemic in this area. We are conducting a research study among contacts of cases of meningitis to test whether a medicine called ciprofloxacin can help to prevent the disease.

We invite your village to participate in this research project because there is an ongoing epidemic in your area. We will explain this document to you. Please ask any questions you have, before you accept or decline to participate on behalf of your village.

**PURPOSE AND PROCEDURES**

Meningitis is a serious infection. It is caused by several different germs, and can be deadly. Once an epidemic has started, the best way to prevent getting meningitis is to be vaccinated. Some vaccines, like MenAfriVac, have been very successful at stopping meningitis in recent years.

Each germ that causes meningitis needs its own specific vaccine to stop cases. And in the last two years, a new germ called “NmC” has appeared. It has caused major epidemics of meningitis in Nigeria and Niger.

Unfortunately, there is a severe shortage of vaccine against NmC around the world, so it has become extremely important to try to find other ways of protecting people against meningitis during an epidemic. One possibility is to use a medicine - an antibiotic - that kills the NmC germ. In this study we will use ciprofloxacin. This antibiotic offers protection for only a few weeks, much shorter than the protection offered by vaccines, but it may be long enough to provide protection in an epidemic.

If you consent that your village participate in the study, it will be randomly assigned to receive one of three interventions when a case is identified in the village. One is a visit from a study nurse who will provide information about meningitis. The second is that household members of the notified case will be offered a single dose of ciprofloxacin. The third is that a village-wide distribution of single-dose ciprofloxacin would be organized after the case has been notified.

All other aspects of medical care of meningitis will remain the same at the health centers and at the district hospital. If someone comes to the health center with symptoms suggestive of meningitis, we will perform testing and treatment following the standard national protocols. If vaccine becomes available, your village would still be eligible to receive the vaccine as well.

During the course of the study, we will look to compare how many cases of meningitis are reported from all of the villages in each of the three arms, and this will allow us to determine which, if any, of the interventions is best for meningitis epidemics in Niger.

It is also possible that your village will be selected to take part in a smaller sub-study where we will collect 3 stool samples from a total of 400 people. We will come to collect the samples over the course of a month and analyse them in the laboratory to ensure that ciprofloxacin is still effective against other germs. We will present a document similar to this one to the people invited to participate in the sub-study.

**RISKS**

Meningitis is a dangerous disease. Untreated, up to half of people who get it may die. Even when treated correctly, the up to 1 person out of 10 will die if they have the disease. So prevention is very important.

Ciprofloxacin is an antibiotic medicine that has been used for many years. It treats many common infections and generally does not cause serious side effects. Children and the elderly may have a slightly increased risk of problems in their tendons if they take ciprofloxacin for a long time, but we are proposing to give only one dose. There is no evidence in humans that suggests it is dangerous to pregnant women or to their unborn children, but there is no sure guarantee of its safety, either. On the other hand, the risks associated with meningitis are quite high for all persons, including pregnant women.

Because it is a strong antibiotic used for many other infections, including some kinds of diarrhea, one of the concerns that we have about using it on a large scale is that it will become less effective against these infections. To make sure, we are performing the sub-study on the stools of 400 persons.

**BENEFITS**

The population of your village may directly benefit from the study interventions. All care you receive will be free of charge. By participating in this study, you may help MSF, Epicentre and the Ministry of Health learn how best prevent cases of meningitis in the future until more doses of vaccine become available.

**CONFIDENTIALITY**

All information collected during this study is strictly confidential. Your name will be recorded on the document we ask you to sign. In the health centers, patient’s names will be collected as usual in the registers, but we will not copy these names. Cases of meningitis will be identified in study databases only with a number. The personal information collected will not be disclosed anywhere outside the study staff. The documents used for the study will be stored in a locked area by Epicentre for 5 years and then destroyed.

**COST/PAYMENT/VOLUNTARY PARTICIPATION**

Your village’s participation in the study is free of charge. You will not be paid for your village’s participation in the study. If you receive a village-wide distribution of ciprofloxacin, it is possible that we will employ some community members to help with the distribution, and they will be paid according to MSF’s standard procedures.

Participation in this study is entirely voluntary. You have a right to refuse your village’s participation or to withdraw participation at any point without negative consequences. If you do not wish to participate in this study, residents of your village will still receive the best possible care for meningitis free of charge in your local health center.

Furthermore, just because you agree that your village be randomized, individuals in your village will not be obliged to participate in the intervention. If they are eligible for treatment with ciprofloxacin, we will explain the nature of the study, the risks and benefits of treatment with ciprofloxacin, and the voluntary nature of participation to the members of the community.

**USE OF THE RESULTS**

At the end of the study, you and other local leaders will be invited to a meeting where the overall results will be presented, without mentioning specific results from any participant. You will be asked to provide a feed-back of this meeting to all the community members. The findings from this study may also be published for use in Niger and in other countries, and we may share our results with decision-makers, but you will never be identified by name.

**IMPLICATION OF YOUR SIGNATURE OR MARK**

If you give your permission for your village to participate in this study, you should sign or make a mark in the permission form. Your signature or mark means that you understand the information given to you about your village’s participation, that the information was explained to you, and that your consent to participate is freely given. You will be asked to sign two copies - one for you to keep and one for the confidential records. Thank you.

For all questions, please contact:

Dr. Oumar Touré, Field Investigator +227 92199440

**Permission for participation document for village chiefs:** **Cluster-randomized trial of ciprofloxacin for contacts of cases of meningococcal meningitis as an epidemic response**

*I* , ……………………………................ *declare that I have read the foregoing information, or it has been read to me. I have had the opportunity to ask questions about it and any questions that I have asked have been answered to my satisfaction. I have understood the objectives and purposes of this study, as well the as the risks and benefits of participation in this study.*

*I consent voluntarily that my village participate in this study and understand that I have the right to withdraw from the study at any time without any consequences for my village.*

**Name of the village:** _________________________________

**Name of the village chief:** _________________________________

**Signature/mark of the village chief:**

Date : ___ ___ / ___ ___ / 20___ ___

day / month / year

**Name of the person obtaining consent:** _________________________________

**Signature of the person obtaining consent:**

Date : ___ ___ / ___ ___ / 20___ ___

day / month / year

***In case of witnessed consent (if the participant is illiterate)***

*I attest that the information in the information sheet and consent form was accurately explained to the above mentioned village chief. I confirm that the village chief has had the opportunity to ask questions, and that he/she freely gave informed consent.*

**Name of the witness:** _________________________________

**Signature of the witness:**

Date : ___ ___ / ___ ___ / 20___ ___

day / month / year

**Permission for participation document for health area officials:** **Cluster-randomized trial of ciprofloxacin for contacts of cases of meningococcal meningitis as an epidemic response**

*I* , ……………………………................ *declare that I have read the foregoing information, or it has been read to me. I have had the opportunity to ask questions about it and any questions that I have asked have been answered to my satisfaction. I have understood the objectives and purposes of this study, as well the as the risks and benefits of participation in this study.*

*I consent voluntarily that the health area for which I am responsible participate in this study and understand that I have the right to withdraw from the study at any time without any consequences for my village.*

**Name/title of the official:** _________________________________

**Signature/mark of the official:**

Date : ___ ___ / ___ ___ / 20___ ___

day / month / year

**Name of the person obtaining consent:** _________________________________

**Signature of the person obtaining consent:**

Date : ___ ___ / ___ ___ / 20___ ___

day / month / year

***In case of witnessed consent (if the participant is illiterate)***

*I attest that the information in the information sheet and consent form was accurately explained to the above mentioned official. I confirm that the official has had the opportunity to ask questions, and that he/she freely gave informed consent.*

**Name of the witness:** _________________________________

**Signature of the witness:**

Date : ___ ___ / ___ ___ / 20___ ___

day / month / year

**APPENDIX B: Informational notice for persons eligible to receive ciprofloxacin**

Madam, Sir,

Médecins Sans Frontières is an independent, medical humanitarian organization providing health care in Niger. Epicentre conducts research in Niger at the request of MSF, the Ministry of Health and other partners in Niger. We are currently supporting the Ministry of Public Health in its response to an ongoing meningitis epidemic in this area. We are conducting a research study among contacts of cases of meningitis to test the efficacy of a medicine called ciprofloxacin in preventing cases.

Your village leaders have given their permission that the village participates in the overall study. As a part of the study, you are eligible to receive ciprofloxacin, but you are under no obligation to do so. We will explain the risks and benefits of taking this medicine, as well as what your participation entails. Please ask any questions you have before you accept or decline to take the ciprofloxacin.

**PURPOSE AND PROCEDURES**

Meningitis is a serious infection. It is caused by several different germs, and can be fatal. Once an epidemic has started, the best way to prevent getting meningitis is to be vaccinated. Some vaccines, like MenAfriVac, have been very successful at stopping meningitis in recent years.

Each germ that causes meningitis needs its own specific vaccine to stop cases. And in the last two years, a new germ called “NmC” has appeared. It has caused major epidemics of meningitis in Nigeria and Niger.

Unfortunately, there is a severe shortage of vaccine against NmC around the world, so we are trying to find other ways of protecting people against meningitis during an epidemic. One possibility is to use an antibiotic that kills the NmC germ. In this study we will use ciprofloxacin. This antibiotic offers protection for only a few weeks, much shorter than the protection offered by vaccines, but it may be long enough to provide protection in an epidemic.

Regardless of whether you take the ciprofloxacin, all other aspects of medical care of meningitis will remain the same at your health center and at the district hospital. If you comes to the health center with symptoms suggestive of meningitis, we will perform testing and treatment following the standard national protocols. And if vaccine becomes available, you would still be eligible to receive the vaccine as well.

During this study, we will look to compare how many cases of meningitis are reported from villages that have different strategies for the distribution of ciprofloxacin. This will allow us to determine which, if any, of the strategies is best for meningitis epidemics in Niger.

**RISKS**

Meningitis is a dangerous disease. Untreated, up to half of people who get it may die. Even when treated correctly, the up to 1 person out of 10 will die if they have the disease. So prevention is very important.

Ciprofloxacin is an antibiotic medicine that has been used for many years. It treats many common infections and generally does not cause serious side effects. Children and the elderly may have a slightly increased risk of problems in their tendons if they take ciprofloxacin for a long time, but we are proposing to give only one dose. There is no evidence in humans that suggests it is dangerous to pregnant women, whereas the risks associated with meningitis are quite high for all persons, including pregnant women.

Because it is a strong antibiotic used for many other infections, including some kinds of diarrhea, one of the concerns that we have about using it on a large scale is that it will become less effective against these infections. To make sure, we are performing a second study on the stools of 400 persons to see if this is the case.

Generally speaking, the risks associated with a single dose of ciprofloxacin seem to be outweighed by the potential benefit.

**BENEFITS**

You may directly benefit from the receiving ciprofloxacin if it is successful at preventing meningitis. All medical care you receive for meningitis will be free of charge. By participating in this study, you may help MSF, Epicentre and the Ministry of Health of Niger learn how best prevent cases of meningitis in the future until more doses of vaccine become available.

**CONFIDENTIALITY**

All information collected during this study is strictly confidential, and we will not ask for your name if you take the ciprofloxacin. In the health centers, patient’s names will be collected as usual in the registers, but we will not copy these names for the study. Cases of meningitis will be identified in study databases only with a number. The personal information collected will not be disclosed anywhere outside the study staff. The documents used for the study will be stored in a locked area by Epicentre for 5 years and then destroyed.

**COST/PAYMENT/VOLUNTARY PARTICIPATION**

Ciprofloxacin will be given free of charge, and you will not be paid for your participation in the study.

Taking the ciprofloxacin is entirely voluntary. You have a right to not take it without negative consequences. If you do not take ciprofloxacin, even if you become sick, you will still receive the best possible care for meningitis free of charge in your local health center.

**USE OF THE RESULTS**

At the end of the study, we will present the results of the study to local leaders, without mentioning specific results from any participant. We will ask them to provide a feed-back of this meeting to you and all other community members. The findings from this study may also be published for use in Niger and in other countries, and we may share our results with decision-makers, but you will never be identified by name.

For all questions, please contact:

Dr. Oumar Touré, Field Investigator +227 92199440

**APPENDIX C: Informational notice and consent document for resistance sub-study**

**Informational notice for participants: Cluster-randomized trial of ciprofloxacin for contacts of cases of meningococcal meningitis as an epidemic response – Resistance sub-study**

Madam, Sir,

Médecins Sans Frontières is an independent, medical humanitarian organization providing health care in Niger. Epicentre conducts research in Niger at the request of MSF, the Ministry of Health and other partners in Niger. We are currently supporting the Ministry of Public Health in its response to an ongoing meningitis epidemic in this area. We are conducting a research study among contacts of cases of meningitis to test the efficacy of a medicine called ciprofloxacin in preventing cases.

We invite you to participate in a part of this research study. We will explain this document to you. Please ask any questions you have, before you accept or decline to participate.

**PURPOSE AND PROCEDURES**

Meningitis is a serious infection. It is caused by several different germs, and can be quite serious. Each germ that causes meningitis needs its own specific vaccine to stop cases, but in the last two years, a new germ called “NmC” has appeared. There is a severe shortage of vaccine against NmC around the world, so it has become extremely important to try to find other ways of protecting people against meningitis during an epidemic.

We are currently conducting a study that looks at how well ciprofloxacin, an antibiotic medicine, works at preventing cases of meningitis among people who have been close to declared cases. But one of the risks about using ciprofloxacin in this way is that it might become less powerful against other infections, including certain kinds of diarrhea.

The sub-study that we are asking you to participate in will compare the kinds of germs living in the stools of people in areas that do not receive ciprofloxacin with the kinds of germs living in the stools of people in areas that do receive ciprofloxacin. We expect that this study will allow us to know whether or not giving ciprofloxacin for meningitis prevention makes this drug less powerful against other germs.

If you consent to participate in the study, we will ask you to provide a sample of your stools on three separate occasions. One today, one in one week’s time, and one in one month’s time. We will provide you with a container and instructions on how to safely collect a sample of your stools (or your child’s stools). We will then arrange to come to the village and collect all the samples at the same time. Before each collection date, we will visit you and give you the information again.

All other aspects of your medical care will remain the same at the health centers and at the district hospital. If you are currently having diarrhea, we will still ask for a sample of your stool, and we will provide you with transportation to the nearest health center for consultation.

After we collect your stool, we will transfer it to the Epicentre laboratory in Maradi. There, we will take a small amount of the stool and test for the presence of germs that are normally treated with ciprofloxacin. Your stool sample will be destroyed within 3 months of its processing and will not be used for any other purposes.

**RISKS**

The risks associated with your participation in the study are expected to be minimal. The main risk is that your hands become soiled with your stool while you place it in the collection container. If this happens, and you do not wash your hands with soap and water, you could place yourself and others at risk of diarrhea. We will provide you with soap each time we give you a stool collection container to ensure that you are able to protect yourself appropriately.

**BENEFITS**

You will not directly benefit from your participation in the study. All care you receive will be free of charge. By participating in this study, you may help MSF learn how best prevent cases of meningitis in the future if no more doses of vaccine become available.

**CONFIDENTIALITY**

All information collected during this study is strictly confidential. Your name will be recorded on the document we ask you to sign and linked to an identification number. On all study documents, you will be identified only by this number and your information will not be disclosed anywhere outside the study staff. The documents used for the study will be stored in a locked area by Epicentre for 5 years and then destroyed.

**COST/PAYMENT/VOLUNTARY PARTICIPATION**

Your participation in the study is free of charge. You will not be paid for your participation in the study, but we will give you soap before each stool sample collection.

Participation in this study is entirely voluntary. You have a right to refuse participation or to withdraw participation at any point without negative consequences. If you do not wish to participate in this study, you will still receive the best possible care free of charge in your local health center.

**USE OF THE RESULTS**

If at any point in the study, we discover that you have a dangerous germ in your stools that is causing you to be sick, we will provide appropriate care free of charge. At the end of the study, your local leaders will be invited to a meeting where the overall results will be presented, without mentioning specific results from any participant. They will be asked to provide a feed-back of this meeting to all of their community members. The findings from this study may also be published by MSF for use in this country and in other countries, but you will never be identified by name.

**IMPLICATION OF YOUR SIGNATURE OR MARK**

If you consent to participate in this study, you should sign or make a mark in the consent form. Your signature or mark means that you understand the information given to you about your participation, that the information was explained to you, and that your consent to participate is freely given. You will be asked to sign two copies - one for you to keep and one for the confidential records.

Thank you.

For all questions, please contact:

Dr. Oumar Touré, Field Investigator +227 92199440

**Informed Consent Document for adult participants:** **Cluster-randomized trial of ciprofloxacin for contacts of cases of meningococcal meningitis as an epidemic response - Resistance sub-study**

*I* , ……………………………................ *declare that I have read the foregoing information, or it has been read to me. I have had the opportunity to ask questions about it and any questions that I have asked have been answered to my satisfaction. I have understood the objectives and purposes of this study, as well the as the risks and benefits of my participation in this study.*

*I consent voluntarily to participate in this study and understand that I have the right to withdraw from the study at any time without any consequences.*

**Name of the participant:** _________________________________

**Signature/mark of the participant:**

Date : ___ ___ / ___ ___ / 20___ ___

day / month / year

**Name of the person obtaining consent:** _________________________________

**Signature of the person obtaining consent:**

Date : ___ ___ / ___ ___ / 20___ ___

day / month / year

***In case of witnessed consent (if the participant is illiterate)***

*I attest that the information in the information sheet and consent form was accurately explained to the above mentioned participant. I confirm that the participant has had the opportunity to ask questions, and that he/she freely gave informed consent.*

**Name of the witness:** _________________________________

**Signature of the witness:**

Date : ___ ___ / ___ ___ / 20___ ___

day / month / year

**Informed Consent Document for child participants:** **Cluster-randomized trial of ciprofloxacin for contacts of cases of meningococcal meningitis as an epidemic response – Resistance sub-study**

*I* , ……………………………................ *declare that I have read the foregoing information, or it has been read to me. I have had the opportunity to ask questions about it and any questions that I have asked have been answered to my satisfaction. I have understood the objectives and purposes of this study, as well the as the risks and benefits of participation in this study.*

*I consent voluntarily that my child* ……………………………................  *participate in this study and understand that I have the right to withdraw my consent from participation in the study at any time without any consequences for me or my child*

**Name of the person providing consent:** _________________________________

**Relation to the participant:** _________________________________

**Signature/mark of the person providing consent:**

Date : ___ ___ / ___ ___ / 20___ ___

day / month / year

**Name of the person obtaining consent:** _________________________________

**Signature of the person obtaining consent:**

Date : ___ ___ / ___ ___ / 20___ ___

day / month / year

***In case of witnessed consent (if the person providing consent is illiterate)***

*I attest that the information in the information sheet and consent form was accurately explained to the above mentioned person. I confirm that the person providing consent has had the opportunity to ask questions, and that he/she freely gave informed consent.*

**Name of the witness:** _________________________________

**Signature of the witness:**

Date : ___ ___ / ___ ___ / 20___ ___

day / month / year

**APPENDIX D: Scientific Committee terms of reference**

**Cluster-randomized trial of ciprofloxacin for prevention of meningitis, Niger**

**Scientific Committee**

**Background**

Médecins Sans Frontières – Geneva Operational Center (MSF-OCG) has been active in Niger for over 10 years. In recent years, it has supported the Ministry of Public Health in its response to seasonal epidemics of meningococcal meningitis. Historically, large epidemics were caused by *Neisseria meningitidis* serogroup A (NmA), but this organism has virtually disappeared since the introduction of a conjugate vaccine (MenAfriVac) in 2010.

The emergence in 2015 of *Neisseria meningitidis* serogroup C (NmC) as an organism capable of causing large-scale epidemics has caused a difficult situation. Available quantities of vaccine containing antigens against NmC are extremely limited, with only about 1.4 million doses available in January 2016 for the entire African meningitis belt.

Because of this unfortunate situation, the WHO has recommended considering novel methods of prevention of meningitis during epidemics in 2016. A cluster-randomized trial testing the impact of single-dose ciprofloxacin for contacts of cases has been planned by Epicentre and MSF-OCG.

**Roles and Responsibilities**

- Drafting of the protocol, trial implementation, analysis of result and the writing of reports and articles will be the responsibility of Epicentre (the primary sponsor of the study), under the responsibility of the study director and the principal investigator in consultation with MSF-OCG (the secondary sponsor and primary financer of the study).
- An external, independent study monitor will be responsible for assuring adherence to study protocols.
- A scientific committee will also be constituted. This document sets forth the terms of reference for this group.

**Scientific Committee Terms of Reference**

The scientific committee is an advisory committee, constituted at the request of Epicentre and MSF-OCG. Its members will have experience in the conduct of clinical trials, MSF operations, laboratory science, and/or the management of meningitis outbreaks. Its members will be selected by Epicentre and MSF-OCG. Members will be invited to participate as independent individuals, and not as representatives of institutions. Members will be asked to declare potential conflicts of interests.

Scientific committee members:

1) Will be asked to comment on a draft version of the protocol. They will be asked to submit their individual comments in writing; a teleconference will be organized thereafter if necessary.

2) Will receive an interim report during the study

3) Will be sent a draft of the final study report for their comments, with a teleconference organized for discussion.

There will be no payment, and the committee will have solely and advisory function.

**Calendar of activities**

The drafting of the protocol will be finalized in the first week of 2016, and the study will begin as soon as possible in an appropriate epidemic zone. Data collection is anticipated through May 2016, with results and analysis provided as soon as possible thereafter. It is anticipated that most of the committee’s work will be performed by email and telephone.

**APPENDIX E: Declaration of Helsinki**

Adopted by the 18th WMA General Assembly, Helsinki, Finland, June 1964

and amended by the:

29th WMA General Assembly, Tokyo, Japan, October 1975

35th WMA General Assembly, Venice, Italy, October 1983

41st WMA General Assembly, Hong Kong, September 1989

48th WMA General Assembly, Somerset West, Republic of South Africa, October 1996

52nd WMA General Assembly, Edinburgh, Scotland, October 2000

53rd WMA General Assembly, Washington DC, USA, October 2002 (Note of Clarification added)

55th WMA General Assembly, Tokyo, Japan, October 2004 (Note of Clarification added)

59th WMA General Assembly, Seoul, Republic of Korea, October 2008

64th WMA General Assembly, Fortaleza, Brazil, October 2013

**Preamble**

1. The World Medical Association (WMA) has developed the Declaration of Helsinki as a statement of ethical principles for medical research involving human subjects, including research on identifiable human material and data.

The Declaration is intended to be read as a whole and each of its constituent paragraphs should be applied with consideration of all other relevant paragraphs.

2. Consistent with the mandate of the WMA, the Declaration is addressed primarily to physicians. The WMA encourages others who are involved in medical research involving human subjects to adopt these principles.

**General Principles**

3. The Declaration of Geneva of the WMA binds the physician with the words, “The health of my patient will be my first consideration,” and the International Code of Medical Ethics declares that, “A physician shall act in the patient's best interest when providing medical care.”

4. It is the duty of the physician to promote and safeguard the health, well-being and rights of patients, including those who are involved in medical research. The physician's knowledge and conscience are dedicated to the fulfilment of this duty.

5. Medical progress is based on research that ultimately must include studies involving human subjects.

6. The primary purpose of medical research involving human subjects is to understand the causes, development and effects of diseases and improve preventive, diagnostic and therapeutic interventions (methods, procedures and treatments). Even the best proven interventions must be evaluated continually through research for their safety, effectiveness, efficiency, accessibility and quality.

7. Medical research is subject to ethical standards that promote and ensure respect for all human subjects and protect their health and rights.

8. While the primary purpose of medical research is to generate new knowledge, this goal can never take precedence over the rights and interests of individual research subjects.

9. It is the duty of physicians who are involved in medical research to protect the life, health, dignity, integrity, right to self-determination, privacy, and confidentiality of personal information of research subjects. The responsibility for the protection of research subjects must always rest with the physician or other health care professionals and never with the research subjects, even though they have given consent.

10. Physicians must consider the ethical, legal and regulatory norms and standards for research involving human subjects in their own countries as well as applicable international norms and standards. No national or international ethical, legal or regulatory requirement should reduce or eliminate any of the protections for research subjects set forth in this Declaration.

11. Medical research should be conducted in a manner that minimises possible harm to the environment.

12. Medical research involving human subjects must be conducted only by individuals with the appropriate ethics and scientific education, training and qualifications. Research on patients or healthy volunteers requires the supervision of a competent and appropriately qualified physician or other health care professional.

13. Groups that are underrepresented in medical research should be provided appropriate access to participation in research.

14. Physicians who combine medical research with medical care should involve their patients in research only to the extent that this is justified by its potential preventive, diagnostic or therapeutic value and if the physician has good reason to believe that participation in the research study will not adversely affect the health of the patients who serve as research subjects.

15. Appropriate compensation and treatment for subjects who are harmed as a result of participating in research must be ensured.

**Risks, Burdens and Benefits**

16. In medical practice and in medical research, most interventions involve risks and burdens.

Medical research involving human subjects may only be conducted if the importance of the objective outweighs the risks and burdens to the research subjects.

17. All medical research involving human subjects must be preceded by careful assessment of predictable risks and burdens to the individuals and groups involved in the research in comparison with foreseeable benefits to them and to other individuals or groups affected by the condition under investigation.

Measures to minimise the risks must be implemented. The risks must be continuously monitored, assessed and documented by the researcher.

18. Physicians may not be involved in a research study involving human subjects unless they are confident that the risks have been adequately assessed and can be satisfactorily managed.

When the risks are found to outweigh the potential benefits or when there is conclusive proof of definitive outcomes, physicians must assess whether to continue, modify or immediately stop the study.

**Vulnerable Groups and Individuals**

19. Some groups and individuals are particularly vulnerable and may have an increased likelihood of being wronged or of incurring additional harm.

All vulnerable groups and individuals should receive specifically considered protection.

20. Medical research with a vulnerable group is only justified if the research is responsive to the health needs or priorities of this group and the research cannot be carried out in a non-vulnerable group. In addition, this group should stand to benefit from the knowledge, practices or interventions that result from the research.

**Scientific Requirements and Research Protocols**

21. Medical research involving human subjects must conform to generally accepted scientific principles, be based on a thorough knowledge of the scientific literature, other relevant sources of information, and adequate laboratory and, as appropriate, animal experimentation. The welfare of animals used for research must be respected.

22. The design and performance of each research study involving human subjects must be clearly described and justified in a research protocol.

The protocol should contain a statement of the ethical considerations involved and should indicate how the principles in this Declaration have been addressed. The protocol should include information regarding funding, sponsors, institutional affiliations, potential conflicts of interest, incentives for subjects and information regarding provisions for treating and/or compensating subjects who are harmed as a consequence of participation in the research study.

In clinical trials, the protocol must also describe appropriate arrangements for post-trial provisions.

**Research Ethics Committees**

23. The research protocol must be submitted for consideration, comment, guidance and approval to the concerned research ethics committee before the study begins. This committee must be transparent in its functioning, must be independent of the researcher, the sponsor and any other undue influence and must be duly qualified. It must take into consideration the laws and regulations of the country or countries in which the research is to be performed as well as applicable international norms and standards but these must not be allowed to reduce or eliminate any of the protections for research subjects set forth in this Declaration.

The committee must have the right to monitor ongoing studies. The researcher must provide monitoring information to the committee, especially information about any serious adverse events. No amendment to the protocol may be made without consideration and approval by the committee. After the end of the study, the researchers must submit a final report to the committee containing a summary of the study’s findings and conclusions.

**Privacy and Confidentiality**

24. Every precaution must be taken to protect the privacy of research subjects and the confidentiality of their personal information.

**Informed Consent**

25. Participation by individuals capable of giving informed consent as subjects in medical research must be voluntary. Although it may be appropriate to consult family members or community leaders, no individual capable of giving informed consent may be enrolled in a research study unless he or she freely agrees.

26. In medical research involving human subjects capable of giving informed consent, each potential subject must be adequately informed of the aims, methods, sources of funding, any possible conflicts of interest, institutional affiliations of the researcher, the anticipated benefits and potential risks of the study and the discomfort it may entail, post-study provisions and any other relevant aspects of the study. The potential subject must be informed of the right to refuse to participate in the study or to withdraw consent to participate at any time without reprisal. Special attention should be given to the specific information needs of individual potential subjects as well as to the methods used to deliver the information.

After ensuring that the potential subject has understood the information, the physician or another appropriately qualified individual must then seek the potential subject’s freely-given informed consent, preferably in writing. If the consent cannot be expressed in writing, the non-written consent must be formally documented and witnessed.

All medical research subjects should be given the option of being informed about the general outcome and results of the study.

27. When seeking informed consent for participation in a research study the physician must be particularly cautious if the potential subject is in a dependent relationship with the physician or may consent under duress. In such situations the informed consent must be sought by an appropriately qualified individual who is completely independent of this relationship.

28. For a potential research subject who is incapable of giving informed consent, the physician must seek informed consent from the legally authorised representative. These individuals must not be included in a research study that has no likelihood of benefit for them unless it is intended to promote the health of the group represented by the potential subject, the research cannot instead be performed with persons capable of providing informed consent, and the research entails only minimal risk and minimal burden.

29. When a potential research subject who is deemed incapable of giving informed consent is able to give assent to decisions about participation in research, the physician must seek that assent in addition to the consent of the legally authorised representative. The potential subject’s dissent should be respected.

30. Research involving subjects who are physically or mentally incapable of giving consent, for example, unconscious patients, may be done only if the physical or mental condition that prevents giving informed consent is a necessary characteristic of the research group. In such circumstances the physician must seek informed consent from the legally authorised representative. If no such representative is available and if the research cannot be delayed, the study may proceed without informed consent provided that the specific reasons for involving subjects with a condition that renders them unable to give informed consent have been stated in the research protocol and the study has been approved by a research ethics committee. Consent to remain in the research must be obtained as soon as possible from the subject or a legally authorised representative.

31. The physician must fully inform the patient which aspects of their care are related to the research. The refusal of a patient to participate in a study or the patient’s decision to withdraw from the study must never adversely affect the patient-physician relationship.

32. For medical research using identifiable human material or data, such as research on material or data contained in biobanks or similar repositories, physicians must seek informed consent for its collection, storage and/or reuse. There may be exceptional situations where consent would be impossible or impracticable to obtain for such research. In such situations the research may be done only after consideration and approval of a research ethics committee.

**Use of Placebo**

33. The benefits, risks, burdens and effectiveness of a new intervention must be tested against those of the best proven intervention(s), except in the following circumstances:

Where no proven intervention exists, the use of placebo, or no intervention, is acceptable; or

Where for compelling and scientifically sound methodological reasons the use of any intervention less effective than the best proven one, the use of placebo, or no intervention is necessary to determine the efficacy or safety of an intervention

and the patients who receive any intervention less effective than the best proven one, placebo, or no intervention will not be subject to additional risks of serious or irreversible harm as a result of not receiving the best proven intervention.

Extreme care must be taken to avoid abuse of this option.

**Post-Trial Provisions**

34. In advance of a clinical trial, sponsors, researchers and host country governments should make provisions for post-trial access for all participants who still need an intervention identified as beneficial in the trial. This information must also be disclosed to participants during the informed consent process.

**Research Registration and Publication and Dissemination of Results**

35. Every research study involving human subjects must be registered in a publicly accessible database before recruitment of the first subject.

36. Researchers, authors, sponsors, editors and publishers all have ethical obligations with regard to the publication and dissemination of the results of research. Researchers have a duty to make publicly available the results of their research on human subjects and are accountable for the completeness and accuracy of their reports. All parties should adhere to accepted guidelines for ethical reporting. Negative and inconclusive as well as positive results must be published or otherwise made publicly available. Sources of funding, institutional affiliations and conflicts of interest must be declared in the publication. Reports of research not in accordance with the principles of this Declaration should not be accepted for publication.

**Unproven Interventions in Clinical Practice**

37. In the treatment of an individual patient, where proven interventions do not exist or other known interventions have been ineffective, the physician, after seeking expert advice, with informed consent from the patient or a legally authorised representative, may use an unproven intervention if in the physician's judgement it offers hope of saving life, re-establishing health or alleviating suffering. This intervention should subsequently be made the object of research, designed to evaluate its safety and efficacy. In all cases, new information must be recorded and, where appropriate, made publicly available.
