# Supplementary material for: Single-dose oral ciprofloxacin prophylaxis as a response to a meningococcal meningitis epidemic in the African meningitis belt: A 3-arm, open-label, cluster-randomized trial
Source: PLoS Med. 2018 Jun 26;15(6):e1002593. doi: 10.1371/journal.pmed.1002593 (PMC6019097; doi:10.1371/journal.pmed.1002593)
Supplement: S1 Table — (DOCX) [file pmed.1002593.s004.docx]

**S1 Table. Ciprofloxacin dosing**

| **Age** | **Dose (mg)** | **Formulation** |
| --- | --- | --- |
| >12 years | 500 | 1 tablet |
| 5-12 years | 250 | 1 tablet |
| 1-4 years | 125 | ½ tablet (250 mg tablet) |
| 3-11 months | 100 | 2 ml oral suspension (250 mg/5ml) |
| <3 months | 75 | 1.5 ml oral suspension (250 mg/5ml) |
