# Supplementary material for: Single-dose oral ciprofloxacin prophylaxis as a response to a meningococcal meningitis epidemic in the African meningitis belt: A 3-arm, open-label, cluster-randomized trial
Source: PLoS Med. 2018 Jun 26;15(6):e1002593. doi: 10.1371/journal.pmed.1002593 (PMC6019097; doi:10.1371/journal.pmed.1002593)
Supplement: S1 Timeline — (DOCX) [file pmed.1002593.s005.docx]

**Protocol / SAP timeline**

*Single-dose oral ciprofloxacin prophylaxis as a response to a meningococcal meningitis epidemic in the African meningitis belt: a three-arm, open-label, cluster-randomized trial*

15 February 2016 – Protocol v1.5 approved by National Consultative Ethics Committee of Niger

31 March 2016 – Trial registered at clinicaltrials.gov (NCT02724046)

29 April 2016 – Protocol v1.5 approved by MSF-ERB

24 March 2017 – Revision of protocol manuscript in *Trials­* – including reviewers’ suggested changes to analytic plan described in protocol v1.5

22 April 2017 – First inclusions in trial

6 June 2017 – Study statistical analysis plan written, in line with published version of protocol in *Trials*

1 July 2017 – Study statistical analysis plan finalized

26 July 2017 – Study database closure
